# Development of a Novel Human Serum Albumin-Based Tool for Effective Drug Discovery: The Investigation of Protein Quality and Immobilization

**DOI:** 10.1021/acs.jmedchem.4c02136

**Published:** 2025-01-15

**Authors:** Balázs Kenéz, Gábor Koplányi, Balázs Decsi, Zsófia Molnár, Péter Horváth, Gábor Katona, György T. Balogh, Diána Balogh-Weiser

**Affiliations:** † Department of Organic Chemistry and Technology, Budapest University of Technology and Economics, Műegyetem rkp. 3, H-1111Budapest, Hungary; ‡ Department of Chemical and Environmental Process Engineering, Budapest University of Technology and Economics, Műegyetem rkp. 3, H-1111Budapest, Hungary; § Department of Pharmaceutical Chemistry, Semmelweis University, Hőgyes E. Street 7−9, H-1092 Budapest, Hungary; ∥ Institute of Pharmaceutical Technology and Regulatory Affairs, Faculty of Pharmacy, University of Szeged, Eötvös u. 6, H-6720 Szeged, Hungary; ⊥ Center for Pharmacology and Drug Research & Development, Semmelweis University, Üllői Street 26, H-1085Budapest, Hungary; # Department of Physical Chemistry and Materials Science, Budapest University of Technology and Economics, Műegyetem rkp. 3, H-1111Budapest, Hungary

## Abstract

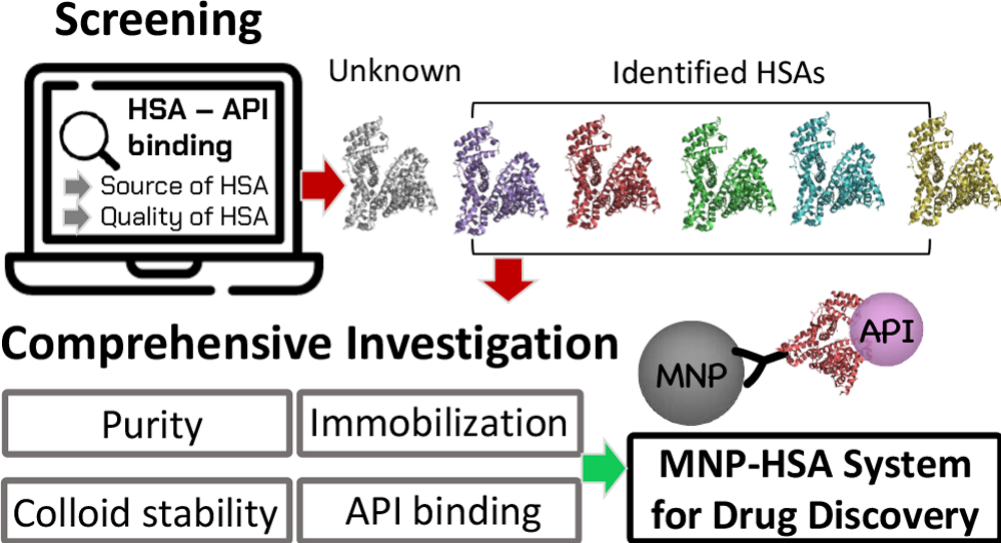

The binding ability of human serum albumin (HSA) on active pharmaceutical ingredients (APIs) is one of the most important parameters in the early stages of drug discovery. In this study, an immobilized HSA-based tool was developed for the rapid and easy in vitro screening of API binding. The work explored the serious incompleteness in the identification of HSA used for in vitro screening published in the last five years. To mitigate this problem, a comprehensive analysis and immobilization studies were performed on the most used HSA types. Serious differences in the colloidal stability of HSAs and their API binding ability on a selected set of APIs were observed. HSAs were immobilized on magnetic nanoparticles with glutardialdehyde (GDA) or cyclohexyl-diglycidyl ether (CDGE) linkers, which have never been used for HSA immobilization before. The HSA-MNP-CDGE complexes achieved a higher immobilization yield and preserved API binding ability; however, the esterase-like enzymatic activity of HSA reduced significantly.

## Introduction

Extensive research has been carried out in the fields of medical sciences, exploring the interactions of proteins with various exogenic and endogenic compounds. Many kinds of interactions have already been studied, with the most relevant in medicine being the inhibition of enzymes and specific protein bindings without catalytic actions.^1^ At the early stages of drug development, the effect of biologically active compounds on enzyme activity is not the only relevant parameter; the compounds’ binding to proteins is also essential. The binding of plasma proteins and drug substances is one of the most important and complex issues, and it has a significant impact on drug bioavailability.^2^ Human serum albumin (HSA) is the most abundant protein in blood plasma. It plays a key function in controlling blood osmotic pressure and transporting various endogenous (such as fatty acids and hormones) and exogenous substances.^3^ For pharmaceutical substances that enter systemic circulation, the binding of the compounds to HSA proteins can change the pharmacokinetics of the drugs, depending on the rate of HSA binding.^4^ The majority of drugs that interact with HSA are anionic; however, a few cationic drugs have also been shown to have detectable affinity for human serum albumin. HSA and medicines interact at two primary sites, which are responsible for binding. Sudlow’s site I (FA7) mediates the binding and transport of bulky heterocyclic anions, whereas Sudlow’s site II (FA3-FA4), which is highly conserved, mediates the binding and transport of aromatic carboxylates in an extended conformation.^5^ HSA is not only a passive but also an active participant in the pharmacokinetic and toxicokinetic processes of endogenous and exogenous substances due to its enzyme-like and noncatalytical activity. Among these, its esterase, peroxidase, and enolase activities are the most relevant, which can change the destiny of active compounds in the human body.^6,7^

In the preclinical stage of pharmaceutical drug research, rapid measurements of drug candidate HSA binding are a critical step in development.^8^ There are many different methods for HSA binding; for example, the most often used is the equilibrium dialysis method,^9^ but HSA-covered high-performance liquid chromatography (HPLC) columns are also highly used. The columns are made by binding the HSA protein to a carrier, most often silica gel or polystyrene, with aldehyde or epoxy function groups. The HSA-HPLC method has the advantage of being quick to measure; it can screen many different compounds simultaneously, and it is continuous, making it easy to connect with analytical devices, for example, mass spectroscopy (MS).^10^ The drawbacks are high cost, limited number of injections, and low pH and solvent tolerance. One of the commonly used equilibrium dialysis methods is rapid equilibrium dialysis (RED), wherein the HSA protein spiked with the analyzed API is loaded on the donor side of the RED cell, and the acceptor side is filled with buffer solution. The cells are shaken and incubated for a few hours at 37 °C; then, an aliquot is taken from both sides, and the drug content is measured with HPLC. The benefits of RED cells are that HSA protein binding can be measured with native HSA proteins, and many samples can be incubated parallelly in a plate system.^11^ The main drawbacks are the long incubation time and the high cost because the RED inserts can only be used once.

**Figure 1:**
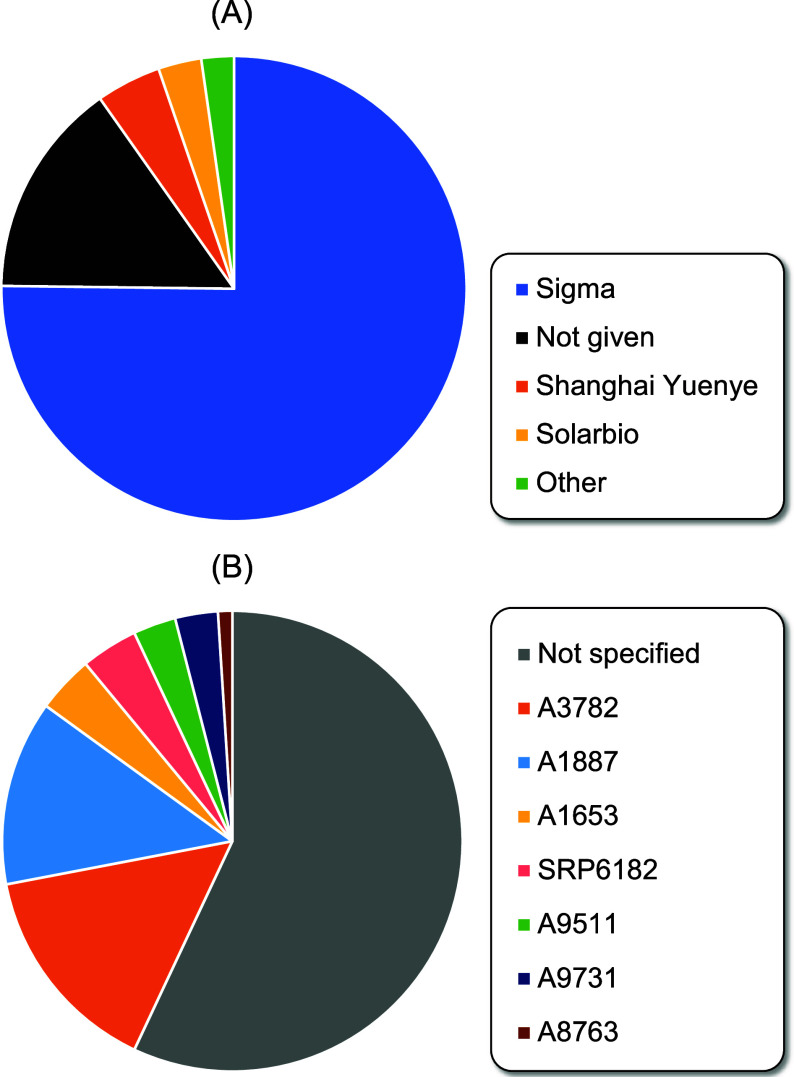
Review of literature data (A) on the most common HSA sources used in HSA-based drug binding studies, from the last 5 years based on the Scopus search engine and (B) most used HSA specified (quality is specified based on purity and the content of excipient) at Sigma-Aldrich.

The specification of HSA is found to be inadequate throughout the literature search for information about the origin and quality of HSA applied for API binding assays. The experimental description either completely or partially omits the source (supplier), purity, and primary excipients, which could cause issues with reproducibility and dependability. To illustrate the problem, scientific papers about HSA binding published in the last five years were compiled using the Scopus database (a detailed list of publications is located in SI, Section 9.). Figure 1A shows the suppliers of the utilized human serum albumin in a pie chart. 100 out of the 133 items could be associated with the Sigma-Aldrich Company. The second largest category was albumins of unknown origin. This happened either because the article claimed that all of the chemicals used were purchased from commercial sources,^12,13^ or because they identified sources but did not specify which source was for which product,^14^ or because they identified the source of substances used for reactions but not for the HSA used for the binding studies.^15^ We also analyzed the various types of used HSA that came from Sigma, and we discovered that out of 100 publications, only 43 had sufficient details about human serum albumin to identify it as a specific product (Figure 1B). This occurred either because they just listed the distributor^16,17^ or because they only provided partial information about the product’s purity (either only purity in percentage based on gel electrophoresis^18^ or type purity, such as fatty acid-free).^19^ Only 18 of the 57 unidentified HSA proteins—roughly one-third of the total—provided at least partial purity data. Regarding the specific types of HSA used in the analyzed literature, A3782 and A1887 were the most used, with 15 and 13 publications, respectively. The most common similarity of these HSA proteins is that they are fatty acid-free (FA) (since FA can strongly influence HSA's structural and functional properties)^20^ and produced from the Fraction V of human serum, A3782 is higher purity and globulin-free as well.

Nanoparticles, due to the continuous improvement of nanotechnology, can open up great possibilities in pharmaceutical applications, and their application in the preclinical stage of drug research has become increasingly important.^21^ Magnetite nanoparticles could be one of the most relevant solid carriers for protein immobilization due to their high chemical resistance, biocompatibility, fine-tunable surface, and ferromagnetic properties, which allow rapid and mild separations.^22,23^ Furthermore, several methods to tailor surface properties allow effective optimization of protein immobilization processes and screening experiments.^24^ The immobilization of HSA onto surface-functionalized bare and core–shell MNPs has already been demonstrated, involving different types of protein–carrier interactions: adsorption,^25^ covalent binding by applying reactive functional groups such as chloro-alkyl,^26^ epoxy,^27^ and aldehyde groups^28^ to form single C–N or double C=N covalent bonds, and primary amino groups to form electrostatic forces^29^ with HSA.

In this work, a novel immobilized HSA-based tool is aimed at developing the rapid and easy screening of HSA-API binding. To achieve this goal, magnetic nanoparticles with the ability of covalent protein binding are produced to ensure the complete and fast separation of HSA from the test mixture. MNP carriers are modified with glutardialdehyde (GDA), the most used covalent linker for HSA immobilization, and an epox-linker, cyclohexyl-diglycidyl ether (CDGE), which had not yet been investigated for HSA binding.

Since our extensive literature search on in vitro binding experiments showed serious incompleteness and inaccuracies in the identification of HSA used for API binding, a comprehensive study of the most used commercially available HSAs (Sigma-Aldrich products such as A9511, A1653, A3782, 126654, and Serva 11877.02) is also being performed. Thus, HSAs from different sources, with different qualities, are systematically compared in the binding of selected drug compounds, covering representative binding sites that have not been investigated so far. The detailed physiochemical characterization of HSAs is also studied by dynamic light scattering, zeta potential, and circular dichroism measurements to explore differences among them and provide new data about commercial HSAs.

The API binding ability of MNP-HAS complexes is also aimed to investigate applying LC-MS detection, and the enzymatic activity of native and immobilized HSAs is examined too by the measurement of their most relevant esterase-like activity, followed by UV–vis spectroscopy assays.

## Results and Discussion

In this study, to demonstrate the significance of serum albumin quality in API binding tests, a detailed comparative investigation was performed involving the most used HSAs from different sources.

### Comprehensive Characterization of Native HSA

**Figure 2:**
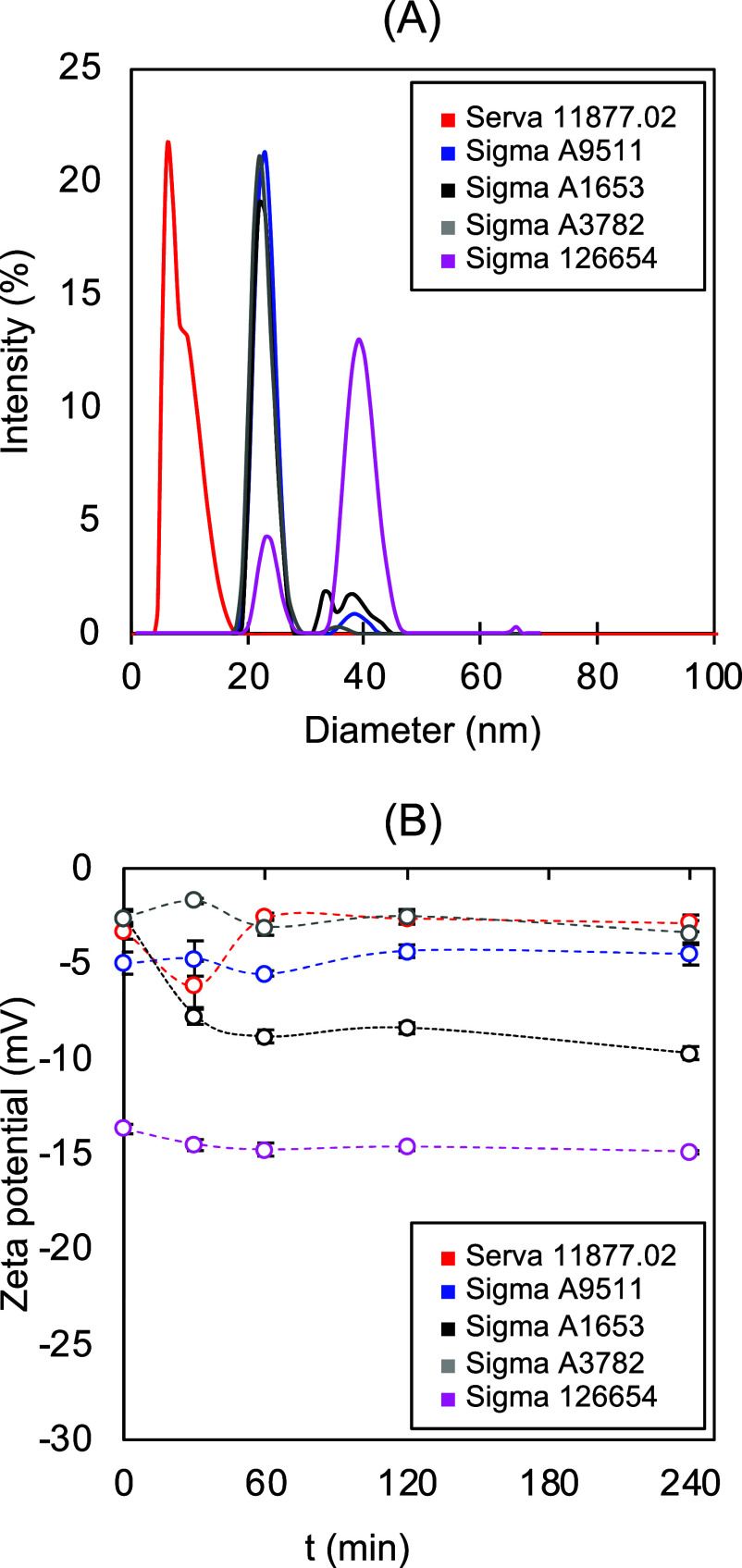
Size distribution (A) and zeta potential (B) of HSA from different sources (Serva 11,877.02, Sigma A9511, Sigma A1653, Sigma A3782, and Sigma 126654) after a 4 h incubation in PBS 50 mM, pH 7.4 at 37.0 °C.

Five different HSA proteins, four of which are Sigma-Aldrich products (A9511, A1653, A3782, and 126654) and one is available at Serva company (11877.02), were analyzed by dynamic light scattering and zeta-potential measurements to explore their colloidal stability and behavior. As can be seen in the DLS results in Figure 2A, there are at least two intensity peaks presented in all of the proteins. The smallest average size is around 10 nm, which is slightly larger than the nominal size of the native HSA molecule (estimated diameter based on protein structure is around 8 nm; PDB: 1AO6). In the case of HSA from Serva, the 10 nm diameter is dominant; however, peak broadening could mean protein aggregation. HSA A9511, A1653, and A3782 from Sigma show an intensity maximum of around 20 nm and some minor peaks at larger sizes. HSA 126654 has stronger aggregation behavior since the population at 40 nm diameter is the most dominant in the sample. Depending on their quality, it could be assumed that the HSAs in solution tend to aggregate. The zeta potentials (*Z*) of the HSAs are measured in order to characterize their colloidal stability (Figure 2B). It is evident that the initial *Z* values vary greatly depending on the type of HSA. Serva 11877.02 and Sigma A3782 have weak stability because they are in an unstable *Z* potential interval (moderately stable interval starts from −5 mV > *Z* > +5 mV). The zeta potential of Sigma A9511 has a more or less constant curve at around −5 mV. The *Z* values of Sigma A1653 became more negative over time, and the most negative *Z* potentials are observed in the case of 126654 HSA (a nondesaturated product of Sigma). Overall, the *Z* potential values of different HSAs do not really change over time, and all HSAs are in the unstable or moderately stable interval, which could mean protein aggregation is in accordance with the DLS results.

Additionally, studies involving sodium dodecyl sulfate polyacrylic amide gel electrophoresis (SDS-PAGE) and circular dichroism spectroscopy (CD) were also performed to characterize HSAs. The SDS-PAGE gel images and the CD spectra do not show significant differences between the HSAs. The albumins are displayed in the 66 kDa range on the SDS-PAGE sheets, while depending on the type of HSA product, different amounts and sizes of contaminants could be observed (see SI, Figure S1). In the CD results, no significant differences are present between the products; among the structural elements, the α helix is the most dominant in all investigated HSAs, as expected (see in SI, Figure S2).

### Investigation of API Binding Applying Native HSA

Following the characterization of the native HSA proteins, the API binding properties were investigated. For a thorough investigation, APIs with different binding sites were selected for the RED cell measurements. The comparative binding data are shown in Table 1, and for comparison, the plasma protein binding of the APIs and HPLC and equilibrium dialysis data from the literature can also be presented. The data show that, in most cases, the binding obtained is lower than plasma protein binding, PPB% (reported in the literature). This can be caused by the presence of other proteins in blood plasma, to which APIs can also bind, sometimes more strongly than the HSA protein. The binding data also proved that the binding of APIs is highly dependent on the level of purity and the method of preparation of HSA involving other components. The most remarkable difference can be noticed in the case of lidocaine, when the only properly detectable binding was achieved with the 126654 HSA. In the case of the other APIs, the closest binding to the literature-reported PPB% was achieved with the A1653 HSA protein, which has the lowest purity among the investigated proteins. It is important to note that some HSA products providing more uniform binding affinity data in the case of API can perform multiple bindings on different sites of HSA. For example, warfarin, azapropazone, and diazepam can bind on sites FA7 and FA3-FA4; thus, they showed a wider range of binding ratios (70.3–86.9%, 88.1–95.4%, and 37.8–62.6%, respectively). In contrast, for more promiscuous active substances in terms of HSA binding, such as diflunisal, indomethacin, thyroxine, and diclofenac, the binding ratio data were observed in a narrower range, with the standard deviation of values obtained by individual HSA isolates typically remaining below 5%. Regarding data heterogeneity, lidocaine was found to be the most interesting among the investigated APIs, since the Cleft (IB)-specific binding could be only achieved with Sigma 126654 and Serva HSAs at moderate binding ratios. However, the HSA binding of lidocaine is weak or moderate, as proven by literature references; the sensitivity of HSA for immobilization could be known from the differences in data about PPB% and IAM-HPLC measurements. Sigma 126654, a nondenaturized HSA, with lidocaine, provided the most similar binding ratio to the referred IAM-HPLC values (23.9% for Sigma 126654 with RED and 20% for IAM-HPLC), but other HSAs from Sigma did not bind lidocaine at all. We can conclude that the affinity of the Cleft binding site depends on not just the immobilization but also the type of HSA is also a key parameter.

**Table 1:** API Binding Properties of Native HSA Measured with RED Cells Compared to the Literature Data (IAM-HPLC or RED)

API	binding site	PPB^30^ (%)	IAM-HPLC or RED^a^	RED				
				Sigma	Sigma	Sigma	Serva	Sigma
				A9514	A1653	126654	11877.02	A3782
warfarin	FA7	99	97.9^3^	70.3 ± 1.8	86.9 ± 0.8	81.9 ± 4.4	84.7 ± 8.1	73.3 ± 1.6
azapropazone	FA7	99	99^a^^31^	88.1 ± 2.1	95.4 ± 2.5	93.1 ± 1.2	93.7 ± 1.8	93.7 ± 3.4
diflunisal	FA3-FA4, FA6, FA7	99	98.7^3^	96.4 ± 0.7	97.4 ± 1.4	96.6 ± 0.5	97.5 ± 0.2	95.9 ± 0.4
indomethacin	FA1, FA7 (partial), FA8	94.5	99.5^3^	90.8 ± 0.4	95.9 ± 1.2	92.0 ± 1.4	92.0 ± 0.9	91.3 ± 0.3
lidocaine	Cleft (IB)	67	20^a^^32^	b	b	23.9 ± 9.2	4.3 ± 19.1	b
thyroxine	Tr-1, Tr-2, Tr-3, Tr-4, Tr-5	99.9	99^33^	94.9 ± 0.8	97.7 ± 0.8	94.9 ± 1.6	96.9 ± 2.0	90.6 ± 0.6
diclofenac	FA1, IIA FA6, FA7	99.5	99^3^	93.4 ± 1.1	97.9 ± 0.9	93.7 ± 3.3	93.7 ± 0.7	96.4 ± 2.2
diazepam	FA3-FA4	99	93.2^3^	59.7 ± 9.8	56.95	37.8 ± 3.0	40.8 ± 8.4	62.6 ± 3.5

a Rapid equilibrium dialysis.

b No discernible difference between the donor and acceptor side, rapid equilibrium dialysis.

### Immobilization of HSA onto Magnetic Nanoparticles

**Figure 3:**
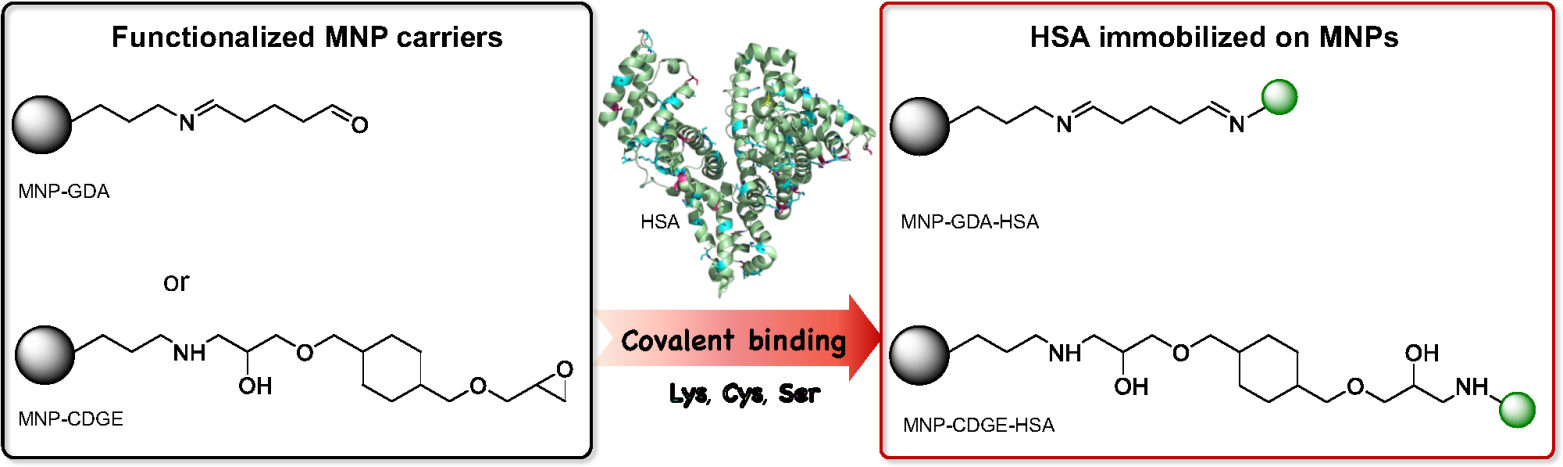
Immobilization strategies for covalent binding of HSA applying functionalized magnetic nanoparticles (MNPs), MNP-GA (MNPs functionalized by glutardialdehyde), and MNP-CGDE (MNPs functionalized by 1,4-cyclohexanedimethanol diglycidyl ether).

Traditional API-HSA binding measurements require a separator tool, for example, a dialysis membrane between the protein-containing phase and the buffer solution, or the immobilization of HSA onto a heterogeneous solid phase. This is the fundamental operating concept of RED systems and HPLC equipped with HSA-filled columns. Both are expensive, and the number of applications is strictly limited. To overcome these challenges, HSA is immobilized on magnetic nanoparticles (MNPs) functionalized with protein binding linkers. MNP carriers have numerous advantageous properties, including ease of separation from the liquid phase by using permanent magnets, good chemical and mechanical resistance, and the ability to modify their surface with various types of functions. In this study, core–shell-structured MNPs (the Fe_3_O_4_ core is covered by a SiO_2_ shell) were produced and then modified with aminopropyltrimethoxysilane to form a primary amine group on the particles. The amino-functionalized MNPs were then reacted with either bisepoxide (1,4-cyclohexanedimethanol diglycidyl ether, CDGE) or glutardialdehyde (GDA) to produce active linkers capable of covalent interactions between the particle and HSA, as shown in Figure 3. GDA is a popular covalent linker or cross-linker in enzyme and protein immobilization methods because it forms Schiff bases with the free amino residues of amino acids, particularly lysine. Several HPLC columns are loaded with HSA bounded onto the solid phase via the GDA linker. Epoxy linkers are also popular, and mainly epichlorohydrin is used for covalent attachment of proteins involving their lysine, serine, or cysteine side chains. Both types of MNPs, MNP-GDA, and MNP-CGDE, were investigated for the covalent binding of the five different HSAs. The immobilization yields (*Y*_I_, %), which were determined using the protein concentration of the initial binding buffer and the protein concentration of the remaining binding buffer after immobilization experiments (*Y*_I_ = 100% means that the total amount of HSA is bound to the MNP surface). Table 2 shows that the bisepoxide linker provides higher immobilization yields for all HSAs compared to the glutardialdehyde linker. Furthermore, *Y*_I_ values demonstrated that the type of HSA has a substantial effect on immobilization. Sigma A1653 and Sigma 126654 were the least attachable to MNPs either in the case of epoxy- or aldehyde-functionalized particles. Serva 11887.02, Sigma A3782, and Sigma A9511 were able to bind similarly to MNP-CGDE, and they showed almost 30% yield of immobilization. Although HSA type Sigma 126654 provided the lowest immobilization yield, *Y*_I_ greater than 10% may indicate an adequate amount of protein for further investigations, and based on its API binding profile, Sigma 126654 was selected for API binding experiments using MNP-HSA.

**Table 2:** Immobilization Yields (*Y*_I_, %) of the Functionalized Magnetic Nanoparticles in Comparison to the Used Linkers (GA or CDGE) and HSA Proteins

MNP carrier	HSA	*Y*_I_(%)
MNP-GA	SERVA 11877.02	7.80 ± 0.6
	Sigma A3782	13.9 ± 9.0
	Sigma A9511	12.1 ± 3.2
	Sigma A1653	a
	Sigma 126654	0.40 ± 0.6
MNP-CDGE	SERVA 11877.02	29.2 ± 6.1
	Sigma A3782	28.6 ± 4.6
	Sigma A9511	28.6 ± 8.3
	Sigma A1653	18.7 ± 0.9
	Sigma 126654	15.4 ± 4.9

a No detectable protein immobilization.

### Investigation of the API Binding of MNP-HSA

**Figure 4:**
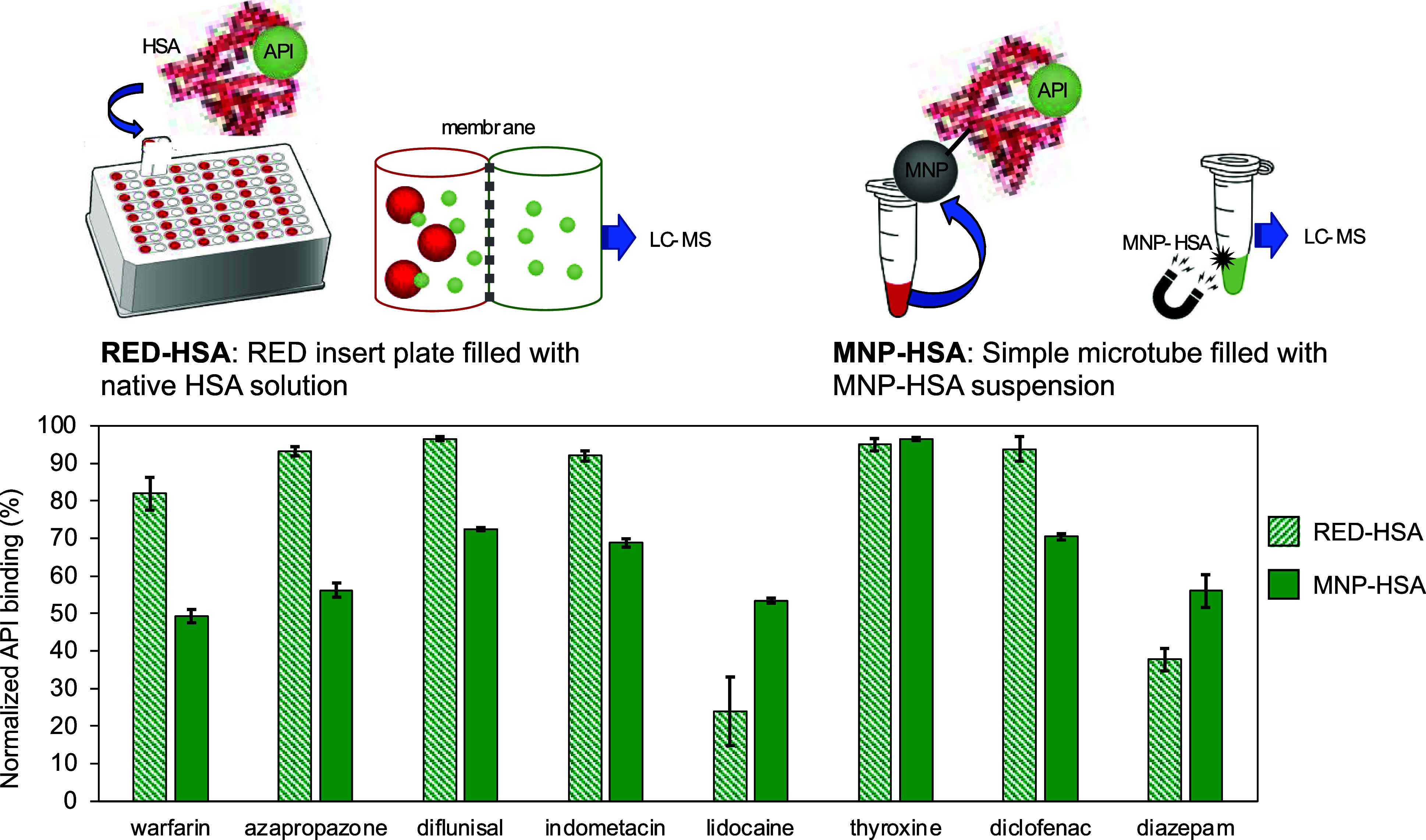
Comparison of HSA-API binding applying RED-HSA (native HSA and API solution are added to a rapid equilibrium dialysis-based insert plate) or MNP-HSA (HSA is covalently immobilized to MNP-CDGE nanoparticles) set ups, using HSA type 126654, incubation for 1 h at 37 °C.

The MNP-HSA API binding was measured by shaking and incubating for 4.5 h. During the incubation time, five samples were collected, and the API concentrations were measured to investigate the binding kinetics of the MNP-HSA systems, which show that the binding usually achieves equilibrium in about one hour. Due to the limitations in the dispersion of the MNPs, in the case of the MNP-HSA measurement setups, lower amounts of protein were used compared to the RED cells. To completely compare the RED and immobilized HSA results, the binding data were normalized to the API/HSA ratio of the RED cell measurements. The normalized 1 h binding results are shown in Figure 4. It can be seen that all of the APIs are bound to the immobilized HSA, but the degree of binding was highly dependent on the API. In most cases, the MNP-HSA produced a lower binding compared to the RED results. This can be due to a conformational shift, caused by the immobilization of the protein, which results in the inactivation of specific binding sites. In the case of thyroxine, the HSA protein has a greater capacity than the other APIs; one protein molecule can bind multiple thyroxines, and the API binding has already reached equilibrium at the first test point (at 0.5 h). The differences between the APIs with single or multiple bindings may also be of interest. In the case of warfarin and azapropazone, a more remarkable drop in the binding ratio can be observed, which can be explained by the covalent binding of HSA on the MNP surface. The immobilization of HSA could influence (mainly blocking) the FA7 binding site of HSA. In the case of lidocaine and diazepam, an enhancing effect was observed since the immobilization of HSA did not modify the Cleft and FA3-FA4 sites. A similar effect can be achieved with thyroxine because the binding results with MNP-HSA were quite comparable to the native form; hence, the natural binding behavior was not much altered. Fatty acid (FA)-free HSA binds to T4 at four different sites, namely, thyroxine-1 to thyroxine-4. These four sites are located in the subdomains IIA, IIIA, and IIIB. The thyroxine-binding crevices partially overlap the FA-binding sites 3–4, FA5, and FA7 binding sites, which display double site occupancy; hence, the immobilization has no inhibitory effect on the drug binding.^34^

**Figure 5:**
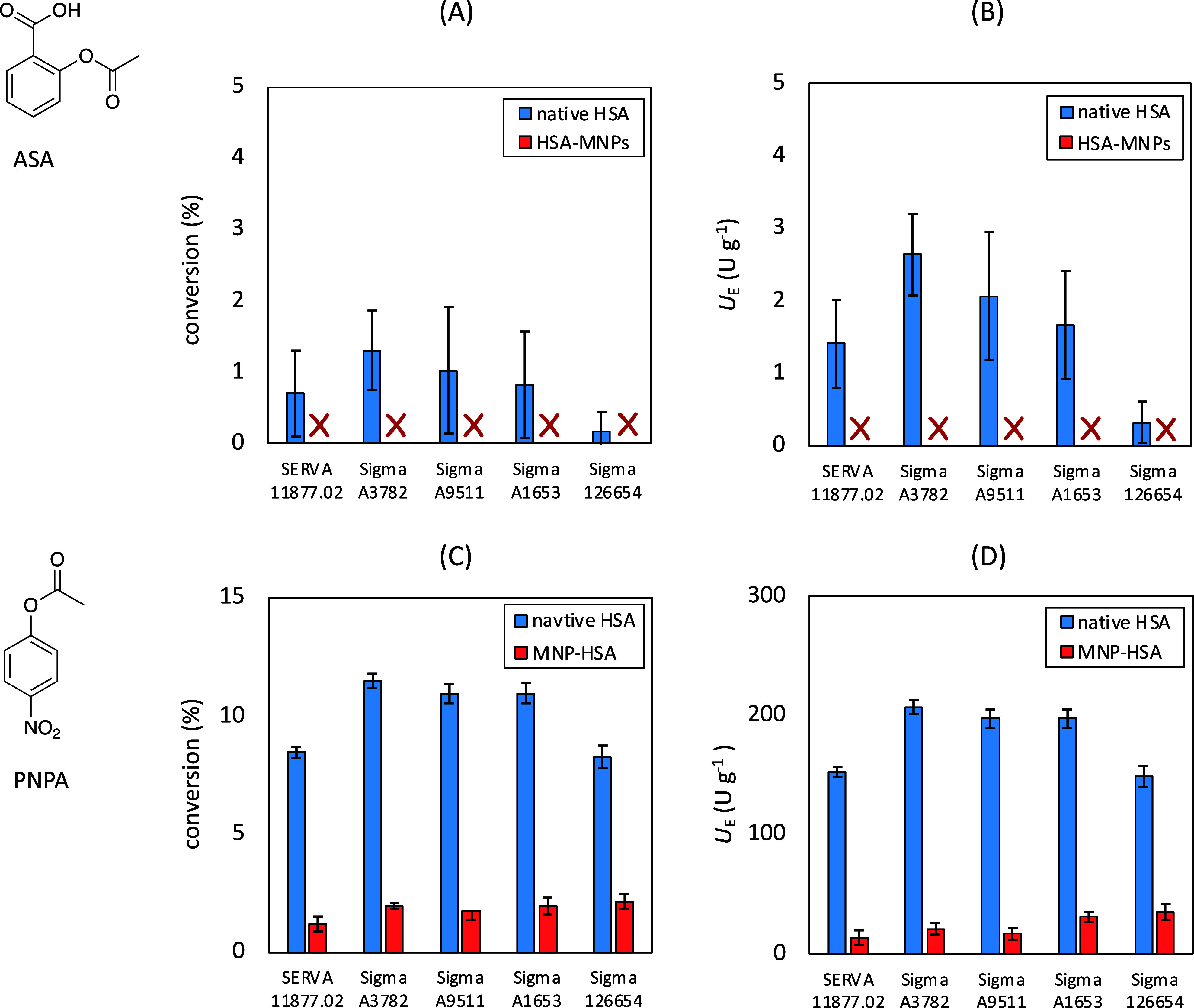
Esterase activity of native HSAs from different origins (Serva 11,877.02, Sigma A3782, Sigma A9511, Sigma A1653, and Sigma 126654) and covalently immobilized HSAs onto magnetic nanoparticles (MNP-CDGE nanoparticles). Conversion values in the HSA-catalyzed biotransformation of aspirin (ASA) (panel A) and *p*-nitrophenyl acetate (PNPA) (panel C) and specific enzymatic activity (*U*_E_) values for substrates ASA (panel B) and PNPA (panel D).

Besides API binding ability, HSA has remarkable enzymatic activities as well. The enzymatic activity could have high importance in API binding assays as well because the transformation of APIs can influence the proper identification of the bounding profile.^35^ Studies showed that HSA has one strong active site and multiple nonspecific catalytic sites located at subdomain IIIA (site II). The active amino acids were suggested to be tyrosine (Tyr411) and histidine residues. Site-directed mutagenesis also proved that Arg410 and Tyr411 are essential for the esterase activity of HSA. Subdomain IIA of HSA (site I) also performs esterase activity, which can convert aspirin (acetylsalicylic acid, ASA) to salicylic acid.^36^ The esterase-like activity of *p*-nitrophenyl esters was also studied. However, it is not only subdomain IIIA that has been assigned to the catalytic activity, since investigations showed that acetylation could not be limited to a distinct side chain so that HSA incubation with high concentrations of PNPA results in the acetylation of 59 Lys, 10 Ser, 8 Thr, 4 Tyr, and 1 Asp residues.^37^ Thus, the esterase activities of different types of native HSAs and HSAs immobilized on CDGE-functionalized magnetic nanoparticles were compared on aspirin (ASA) and *p*-nitrophenyl acetate (PNPA) as model substrates. To evaluate the enzymatic ability of HSA, standard assays were performed based on literature data, and the substrate conversion (conversion, %) was determined. Specific enzymatic activity (*U*_E_, U g^–1^, which shows the amount of product [μmol] generated per unit of time [min] and unit of enzyme amount [g]) was also introduced for the correct comparison of enzymatic assays (see details in Section 8 in the SI). In the case of aspirin, native HSA types showed moderate substrate conversion and enzymatic activity. There were no remarkable differences between Serva and A3782, A9511, and A1653 HSA, and 126654 HSA showed the weakest esterase activity (Figure 5A,B). It is important to note that all HSAs immobilized on CGDE-functionalized magnetic nanoparticles did not show esterase activity on aspirin. Native HSA had much higher catalytic ability on the PNPA substrate since all of them produced a ca. 10% conversion rate and enhanced specific activity (*U*_E_). The activity of immobilized HSA significantly decreased in all cases (Figure 5C,D).

## Conclusions

In this study, a novel HSA protein-based tool was developed for in vitro screening of HSA-API binding. To achieve this goal, a thorough investigation of the HSA protein and covalent immobilization of HSA by applying magnetic nanoparticles was performed. The comprehensive literature search related to HSA-API binding made it obvious that different types of HSA are used, and the identification of HSA (source, quality, and purity) is not given or is incomplete in most experiments, causing serious difficulties in the coherency and reproducibility of binding data. To mitigate this gap, this study provided a detailed characterization of the most popular native HSA proteins used for API binding in the literature. The colloidal stability of the used HSAs was investigated with DLS and zeta-potential measurements, which showed that the quality and purity of the protein affect the solution stability of the HSA, while the incubation time had no significant effect. HSAs were also investigated with CD measurements and SDS-PAGE analysis, which showed no significant differences between the different types of protein. The API binding ability of the native HSAs was also investigated by involving a selected set of APIs to represent the specific binding sites of HSA. API binding investigations showed significant differences among the HSAs. In the case of lidocaine, only one, the nondenaturated HSA (126654, Sigma), showed binding ability, which correlates with the in vivo data.

The HSAs were immobilized on the magnetic nanoparticles' surface modified with a bisepoxide linker (CGDE), which was never used for HSA immobilization before, or dialdehyde (GDA) as the most used linker capable of covalent binding. Based on the immobilization yield results, the bisepoxide linker had higher immobilization efficiency compared to GDA. The API binding of MNP-HSA complexes was also investigated, and the kinetics of the binding showed that equilibrium was reached around the 1 h time frame. The MNP-HSA complexes had successful API binding ability; however, the total binding was lower in comparison to RED measurements by applying native HSA, caused by lower protein concentrations of the MNP-HSA. In addition, the esterase-like enzymatic activity of different types of HSAs was compared by applying native HSA products and HSA immobilized on magnetic nanoparticles. Results with model substrates, aspirin, and *p*-nitrophenyl acetate, proved that native forms have relevant esterase activity, and the immobilization of the albumin significantly decreased or eliminated the esterase activity. In the case of the API-binding-focused investigation, covalent immobilization on magnetic nanoparticles could mean additional advantages since it can exclude unwanted artifacts in binding profiling.

## Experimental Section

All reagents and compounds used for the experiments are ≥95% pure based on the proper manufacturer's data sheet.

### Immobilization of HSA on MNP

Smaller scale for immobilization yield measurements:

In a 1.5 mL microcentrifuge tube (Eppendorf Safe-Lock), MNP (5.0 mg) and phosphate buffer (PBS, 665 μL, 50 mmol, pH 7.4) were added. The mixture was sonicated in an ultrasonic bath for 15 min, and the HSA protein solution (335 μL, 1.0 mg mL^–1^, PBS, 50 mmol, pH 7.4) was added. The microcentrifuge tube was sealed and shaken at 1100 rpm for 24 h at room temperature. The HSA-containing mixture was separated using a neodymium magnet, the supernatant was sampled, and the HSA protein concentration of the sample was determined using a NanoDrop UV–vis spectrophotometer.

### Native HSA-API Binding Measurements with RED Cells

The API binding of the native HSA protein was measured with a Thermo Scientific Rapid Equilibrium Dialysis (RED) plate and inserts (8 K MWCO). The measurements were performed in triplicate. The measured APIs were kept as 10 mM DMSO solutions and diluted with acetonitrile to 1 mM. In the case of thyroxine, the DMSO solution was freshly made before the measurements, and methanol was used in all cases instead of acetonitrile. From the 1 mM solutions, 27.5 μL was taken and diluted to 1 mL with HSA solutions (4 mg mL^–1^, PBS, 50 mmol, pH 7.4) to make the donor solution. To the red cells was added 200 μL of the donor solution, and to the acceptor side, 350 μL of PBS solution (50 mmol, pH 7.4) was added. The plate was sealed with foil, and the plate was shaken at 37.0 °C and 300 rpm for 4.5 h using an orbital shaker equipped with a thermo-incubator (Vibramax 100, Heidolph Gmbh, Schwabach, Germany). After the incubation, 50 μL of samples was taken from the donor and acceptor sides. To the donor sample, 50 μL of PBS was added, and to the acceptor side, 50 μL of HSA solution (4 mg/mL, PBS, 50 mmol, pH 7.4) was added. The samples were then diluted with 300 μL of B solvent (90:10 acetonitrile: water v/v%, 0.1 v/v% formic acid, 1 μM Verapamil as an internal standard) or methanol in the case of thyroxine. The samples were then incubated on ice for 30 min and then centrifuged at 14,000*g* at 4 °C. From the supernatant, 200 μL was taken and then analyzed with LC-MS. The LC-MS methods can be found in the supporting material.

### API Binding Measurements Applying MNP-HSA

In a 1.5 mL microcentrifuge tube (Eppendorf Safe-Lock), MNP (20.0 mg) and phosphate buffer (PBS, 600 μL, 50 mmol, pH 7.4) were added. The mixture was sonicated in an ultrasonic bath for 10 min. After sonication, the API solutions (12.5 μM, 400 μL, 50 mmol of PBS, pH 7.4) were added to the mixture. The API solution was prepared from 10 mM stock solutions, which were diluted to 1 mM with acetonitrile or methanol in the case of thyroxine. The 1 mM solutions were then diluted with PBS to 12.5 μM. The prepared API-containing mixtures were then shaken at 1100 rpm and 37.0 °C for 4.5 h using an orbital shaker equipped with a thermo-incubator (Vibramax 100, Heidolph Gmbh, Schwabach, Germany). From the mixtures, 20 μL of samples was taken at certain intervals (0.5, 1, 2, 3, and 4.5 h). The samples were diluted with B solvent (90:10 acetonitrile: water v/v%, 0,1 v/v% formic acid) or methanol in the case of thyroxine to 100 μL and then analyzed with LC-MS. The used LC-MS methods can be found in the supporting material.

## Supporting Information Available

Materials and methods, chemical, reagents, and proteins; synthesis of the MNP carriers; functionalization of MNP carriers; SDS-PAGE analysis of HSA; CD measurement of HSA; DLS and zeta-potential measurements of the native HSA proteins; HPLC-MS analysis of HSA-API binding experiments; investigation of enzymatic activity of HSA; and results of the literature search about HSA applied for API binding (PDF)

## Abbreviations

API: active pharmaceutical ingredients
ASA: acetyl salicylic acid
CD: circular dichroism spectroscopy
CGDE: 1,4-cyclohexanedimethanol diglycidyl ether
DLS: dynamic light scattering
FA: fatty acid
GA: glutardialdehyde
HPLC: high-performance liquid chromatography
HSA: human serum albumin
MS: mass spectroscopy
MNP: magnetic nanoparticles
PNPA: p-nitrophenyl acetate
PPB: plasma protein binding
RED: rapid equilibrium dialysis
SDS PAGE: sodium dodecyl sulfate polyacrylic amide gel electrophoresis
