# Supplementary material for: Development of a Novel Human Serum Albumin-Based Tool for Effective Drug Discovery: The Investigation of Protein Quality and Immobilization
Source: J Med Chem. 2025 Jan 15;68(3):2840–8. doi: 10.1021/acs.jmedchem.4c02136 (PMC11831590; doi:10.1021/acs.jmedchem.4c02136)
Supplement: Supplementary file 1 — jm4c02136_si_001.pdf [file jm4c02136_si_001.pdf]

Supporting Information

Development of a Novel Human Serum Albumin Based Tool for Effective Drug Discovery – The investigation of Protein Quality and Immobilization

Balázs Kenéz[1], Gábor Koplányi[1], Balázs Decsi[2], Zsófia Molnár[1], Péter Horváth[3], Gábor Katona[4], György T. Balogh[2,3]*, and Diána Balogh-Weiser[1,5]*

[1] Department of Organic Chemistry and Technology, Budapest University of Technology and Economics,
H-1111, Műegyetem rkp. 3., Budapest, Hungary

[2] Department of Chemical and Environmental Process Engineering, Budapest University of Technology and Economics,
H-1111, Műegyetem rkp. 3., Budapest, Hungary

[3] Department of Pharmaceutical Chemistry, Semmelweis University, Hőgyes E. Street 7–9, H-1092 Budapest, Hungary

[4] Institute of Pharmaceutical Technology and Regulatory Affairs, Faculty of Pharmacy, University of Szeged, Eötvös u. 6,
H-6720 Szeged, Hungary

[5] Center for Pharmacology and Drug Research & Development, Semmelweis University,
H-1085, Üllői Street 26., Budapest, Hungary

[6] Department of Physical Chemistry and Materials Science, Budapest University of Technology and Economics,
H-1111, Műegyetem rkp. 3., Budapest, Hungary

Corresponding Author's email address: balogh.weiser.diana@vbk.bme.hu,
balogh.gyorgy.tibor@semmelweis.hu

1. Chemicals, reagents, and proteins

The HSA proteins that were used in the study, A9511 (>=97% agarose gel, lyophilized powder), A1653 (>=96% agarose gel, remainder mostly globulins, lyophilized powder), A3782 (>=99% agarose gel, essentially fatty acid-free, essentially globulin-free, lyophilized powder), 126654 (>=95% SDS-PAGE, non-denaturized, lyophilized powder) were bought from Sigma-Aldrich Ltd. (St. Louis, MO, USA). The 11877.02 (>=92% total protein, >=97% HSA purity) HSA protein was bought from Serva Electrophoresis LLC (Heidelberg, Germany).

The chemicals used in this study were purchased from the following companies: 1,4-Cyclohexanedimethanol diglycidyl ether (CDGE) was bought from Ipox Chemicals Ltd. (Budapest, Hungary). Sodium-dihydrogen phosphate, tetraethoxysilane (TEOS), ammonium-hydroxide (25% aq and 35% aq.) 3-aminopropyl-trimethoxysilane (ApTMOS), anhydrous sodium-acetate, acetic acid, ethylene-glycol, polyethylene glycol 4000, polyethylene glycol 400 (PEG4000, PEG400) Iron (III)-chloride, glutardialdehyde (GDA, 25 m/m%, aq. solution), acetonitrile (HPLC grade), warfarin, azapropazone, diflunisal, indomethacin, lidocaine, thyroxine, diclofenac, diazepam were bought from Sigma-Aldrich Ltd. (St. Louis, MO, USA).

2. Synthesis of the MNP carriers

The magnetic nanoparticles were prepared by solvothermal synthesis, in which iron (III) chloride (20.2 g) was dissolved in ethylene glycol (600 mL) in a 1-Liter Erlenmeyer flask, which has a ground glass joint, by stirring with a magnetic stirrer. After dissolving the iron chloride, PEG 4000 (20.2 g) and sodium acetate (54.0 g) were added to the solution. The mixture was

then stirred until the components were completely dissolved to give a homogeneous, ochre-yellowish solution. The mixture was poured into a stainless-steel laboratory autoclave, a cross-shaped stir bar was added, the autoclave was carefully sealed, and the system was kept at 200°C for 24 hours with intensive stirring. The heating was stopped after 24 hours, and the autoclave was left to cool down (3-4 hours). Magnetic nanoparticles were isolated from the black suspension formed during the reaction using a neodymium magnet (N45). After the isolation of MNPs from the glycol, the remaining particles are washed out of the autoclave with distillated water. After that, the particles were thoroughly washed three times with ethanol, three times with distilled water, and three times with 2-propanol and dried in a vacuum drying oven (at room temperature, 10 mbar in a Binder VDL 23 vacuum drying chamber, Binder GmbH, Tuttlingen, Germany) until they reached a constant mass.

Then, the silica shell was prepared according to the Stöber-method. MNP (5.0 g) and PEG400 (5.0 g) were measured into a half-Liter Erlenmeyer flask with a ground glass joint and a stopper. After that, abs. Ethanol (125 mL) and distilled water (25 mL) were added. The mixture was sonicated (35 kHz, 160 W in a Sonorex Digitec DT255 US bath, Bandelin Gmbh, Berlin, Germany) for 30 minutes to ensure that the magnetic nanoparticle clusters were properly dispersed. After the sonication, ammonia solution (12.5 mL, 35% aq. NH_4OH) was added, followed by the TEOS precursor (7.5 mL). After the additions, the flask was plugged, covered with parafilm, and put on an orbital shaker (Vibramax 100, Heidolph Gmbh, Schwabach, Germany) for 24 hours (the shaking speed was on the lowest setting). After 24 hours of reaction time, the particles were washed several times with distilled water (until ammonia-free), isolated with a neodymium magnet, then washed three times with ethanol, and dried in a vacuum drying oven (at room temperature, 10 mbar in a Binder VDL 23 vacuum drying chamber, Binder GmbH, Tuttlingen, Germany) until they reached constant mass.

3. Functionalization of MNP carriers

The amino-functionalization of silica-coated MNPs was performed in a 20-mL vial, applying MNP-TEOS (250 mg), PEG 400 (45 µL), and ethanol (2.5 mL). The mixture was sonicated for 30 minutes (35 kHz, 160 W in a Sonorex Digitec DT255 US bath, Bandelin Gmbh, Berlin, Germany). To prepare the solution, ApTMOS (525 µL) was added to a 20-mL vial, and the solution was diluted with ethanol to 5 mL. After the sonication, ammonia solution (12.5 mL, 35% aq. NH_4OH) was added to the mixture. The mixture was shaken for 10 minutes. After the shaking, 5 mL of the prepared ethanolic silane solution was added to the mixture. The flask was sealed and put on an orbital shaker (Vibramax 100, Heidolph Gmbh, Schwabach, Germany) for 24 hours at 400 rpm. After the 24-hour reaction time, the particles were washed three times with ethanol, three times with distillated water, and three times with isopropanol. After the washing, the particles were dried in a vacuum drying oven (at room temperature, 10 mbar in a Binder VDL 23 vacuum drying chamber, Binder GmbH, Tuttlingen, Germany) until they reached a constant mass.

Then, amino-functionalizd MNPs were modified with bifunctional linkers (CDGE or GDA). In a 4 mL-vial, amino-functionalized MNP (25 mg), ethanolic PEG 400 solution (200 µL, 50 mg mL^{-1}), and ethanol (1 mL) were added. The mixture was sonicated for 20 minutes (35 kHz, 160 W in a Sonorex Digitec DT255 US bath, Bandelin Gmbh, Berlin, Germany). After the sonication, CDGE solution (400 µL, 1,5 M ethanolic solution) or GDA solution (225 µL) was added. The flask was sealed, and then the reaction mixture was shaken for 24 hours at 800 rpm using an orbital shaker (Vibramax 100, Heidolph Gmbh, Schwabach, Germany) at 60°C. After the 24-hour reaction time, the particles were washed three times with ethanol. After the washing, the particles were dried in a vacuum drying oven (at room temperature, 10 mbar in a Binder VDL 23 vacuum drying chamber, Binder GmbH, Tuttlingen, Germany) until they reached a constant mass.

4. SDS-PAGE analysis of HSA

The purity and composition of the HSA proteins were tested with SDS-PAGE (sodium dodecyl sulphate polyacrylamide gel electrophoresis). Measurements were performed using an Enduro Power Supplies transformer and BioRad run set. For the SDS-PAGE measurements, polyacrylamide gels were prepared according to the following recipes (for four gel sheets):

- Seperating/resolving gel: 7.35 mL distilled water, 3.75 mL separating buffer (1.5 M TRIS; pH 8.8), 3.75 Ll 40% acrylamide solution, 150 µl 10% SDS solution, 150 µL 10% APS solution and 15 µL TMEDA were added to a 50 ml centrifuge tube.
- Stacking gel: 3.2 mL distilled water, 1.25 mL packing buffer (0.5 M TRIS; pH 6.8), 505 µl 40% acrylamide solution, 50 µl 10% SDS solution, 50 µL APS solution and 10 µL TMEDA were added to a 50 ml centrifuge tube.

The APS solution and TMEDA were added to the gel precursor solutions before casting for both the separating and the stacking gel. Initially, the separating gel was poured between the pre-assembled glass plates. After the polymerization was completed, the compression gel was poured upon the separation gel, a comb was inserted to form the sample site, and the polymerization was allowed to complete.

From the HSA proteins, a solution was prepared (3.7 mg mL^{-1}, 100 mM pH 7.4 potassium phosphate buffer). From the HSA solutions, 20 µL samples were taken into Eppendorf tubes, and 5 µl of sample preparation solution (250 mM Tris-HCl, 6.8 pH, 10% SDS, 10% DTT, 50% glycerol, 0.05% bromophenol blue) was added to each tube and kept in an Eppendorf thermoblock at 95 °C for 5 minutes. Gel plates were placed in the frame of the running bath and filled with running buffer (for 1 liter of buffer: 3.03 g TRIS, 14.4 g glycine, and 1 g SDS). The comb was removed from the gel sheet, and 5 µl or 1 µL of each sample were pipetted into the gel pockets. The first and last pockets of the sheet were filled with 5 µL of ThermoFisher Scientific protein marker. The molecular weights of the ladder proteins are 18.4; 25; 35; 45; 66.2; 116 kDa. The gel electrophoresis was performed at 200 V until the bottom of the running front gel plate was reached. After the electrophoresis the gel was removed from the glass panels and the stacking gel part was cut off. The separating gel was then placed in a plastic container filled with distilled water and it was then heated in the microwave for 10 second, then shaken for 5 minutes. The water was discarded, then the container was filled with ProSieve Blue Protein Staining Solution dye developer and shaken overnight, to stain the proteins in the gel blue. The next day the dye solution was poured off, then the container was filled with distillated water. The container was heated in the microwave for 10 second then shaken for 5 minutes. The stained distillated water was poured off, then fresh distillated water was added to the container. This process was repeated until no more dye washed out of the gel. The photo of the result can be seen in Figure S1.

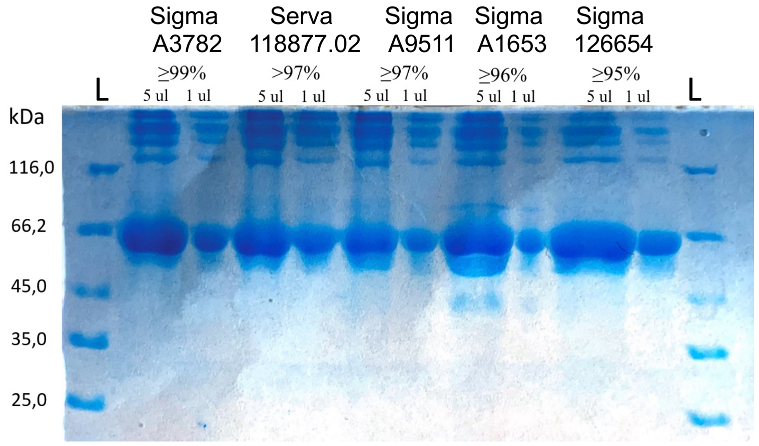

FigS1. SDS PAGE analysis of HSA from different sources (Sigma A3782, Serva 1188877.02, Sigma A9511, Sigma A1653 and Sigma A126654), L is the protein standard ladder.

5. CD measurement of HSA

CD experiments were performed on a Jasco J-815 spectrometer (Jasco LTD, Tokyo, Japan) in Hellma cuvettes with path lengths of 0.1 cm. The slit was set to 2 nm, the registration speed was set to 20 nm min^{-1}, and 3 scans were averaged in each experiment in 250–185 nm wavelength range. The temperature was set to 25.0ºC and 37.0ºC using a Jasco CDF-426L Peltier thermostat. The initial concentration of the HSA stock solution was 1 mg 1 mL^{-1}, and a 100 mL stock solution was diluted with 200 mL of ultrapure water. The secondary structure of proteins was estimated using BeStSel [https://doi.org/10.1093/nar/gkac345] CD analysis software (v1.3.230210). The estimated values of the secondary structural elements of the proteins are shown in the Table S1 below.

Table S1. Distribution of secondary structural elements at 25ºC and 37ºC in percent

Sample	Sigma A1653		Sigma A3782		Sigma A9511		Sigma 126654		Serva 11877	
sec. struct.	25ºC	37ºC	25ºC	37ºC	25ºC	37ºC	25ºC	37ºC	25ºC	37ºC
Helix	50.9	46.6	50.0	48.3	50.8	49.6	48.6	48.6	50.3	49.3
Antiparalell sheet	3.3	3.8	3.8	3.1	5.2	5.3	3.3	6.2	4.5	7.3
Paralell shet	2.3	2.8	2.0	1.9	1.1	2.0	3.7	1.8	1.9	1.3
Turn	11.3	12.2	11.0	12.0	11.3	11.7	11.9	11.3	10.8	11.6
Other	32.23	34.5	33.3	34.7	31.6	31.5	32.5	32.1	32.2	30.4

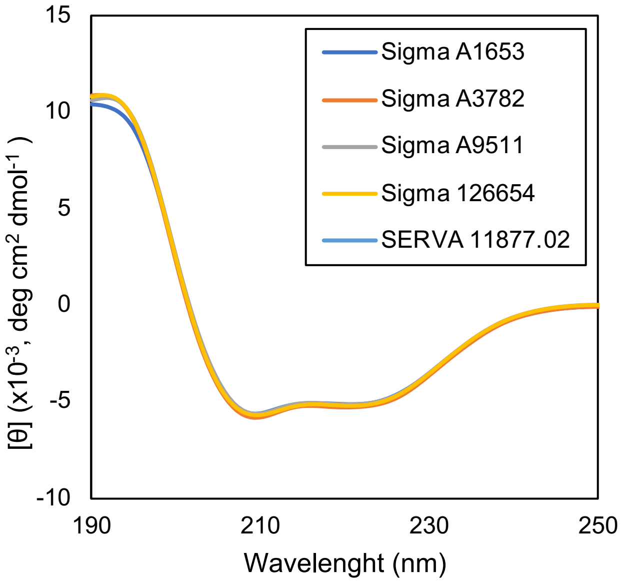

FigS2. CD spectra of HSA from different sources (Sigma A3782, Serva 1188877.02, Sigma A9511, Sigma A1653 and Sigma A126654) in ultrapure water at 37ºC.

6. DLS and Zeta potential measurements of the Native HSA proteins

From the different HSA proteins, a 1 mg mL^{-1} solution was prepared (PBS, 50 mM, pH 7.4) and shaken at 450 rpm for 4 h at room temperature in an orbital shaker (Vibramax 100, Heidolph Gmbh, Schwabach, Germany). Samples were taken at the beginning and after 30 min, 1 h, 2 h, and 4 h. The size distribution of the HSA proteins and the zeta potential of their solutions were measured using Malvern Panalytical Zetasizer Pro Blue (Malvern Pananalytical, Worcestershire, UK) DLS and a zeta potential analysis system. The mean particle size and zeta potential of samples from each protein solution were determined in a 10 x 10 mm PS cuvette (Malvern Panalytical, DTS0012) using a Zetasizer Dip-cell (ZEN1002) from 3 parallel measurements at room temperature using the default method in the instrument software.

7. HPLC-MS analysis of HSA-API binding experiments

The HPLC-MS measurements to determine the HSA-API binding was performed on a Waters 2690 liquid chromatography system (Waters Corporation, Milford, MA, USA) equipped with a Waters 2487 Dual Absorbance diode array detector (DAD, Waters Corporation, Milford, MA, USA) and coupled with a Waters Micromass Quattro Ultima Pt tandem quadrupole mass spectrometer (Waters Corporation, Milford, MA, USA). Chromatographic analysis was performed on a Kinetex® XB 2.6 µm C18 100 Å column (150 × 4.6 mm) at 40 °C. MassLynx 4.0 was used for data acquisition and analysis. Eluent **A** (100% H_2O, 0.1% HCOOH) and Eluent **B** (95:5% AcN:H_2O, 0.1% HCOOH) were applied for the corresponding HPLC-MS method (Methods M1–M3) are detailed in Table S1).

Table S2. HPLC methods applied for the analysis of HSA-API binding experiments.

Method	Flow rate (mL min^{-1})	Gradient elution	Total runtime (min)
M1	1	0 min: 5% B<br>11 min: 100% B<br>13 min: 100% B<br>13.01 min: 5% B	15
M2	1.2	0 min: 20% B<br>11 min: 100% B<br>13 min: 100% B<br>13.01 min 20% B	15
M3	1	0 min: 5% B<br>10 min 50% B<br>11 min: 100% B<br>13 min: 100% B<br>13.01 min: 5% B	15

Table S3. HPLC-MS data of different APIs investigated in HSA binding experiments.

API	Method	Time of retention (min)	Molecular mass (Da)	m/z	Ionization mode
**Azapropazone**	M1	6.09	300.16	301.2	ES+
**Diazepam**	M1	9.22	284.74	285.4	ES+
**Diclofenac**	M1	10.65	296.1	250.1	ES-
**Diflunisal**	M2	10.22	250.2	249.1	ES-
**Indomethacin**	M1	10.27	357.8	358.5	ES+
**Lidocaine**	M3	4.38	234.34	235.2	ES+
**Thyroxine**	M1	7.37	776.87	778.1	ES+
**Verapamil**	M1	6.35	454.6	445.8	ES+
**Warfarin**	M1	9.52	308.3	309.1	ES+

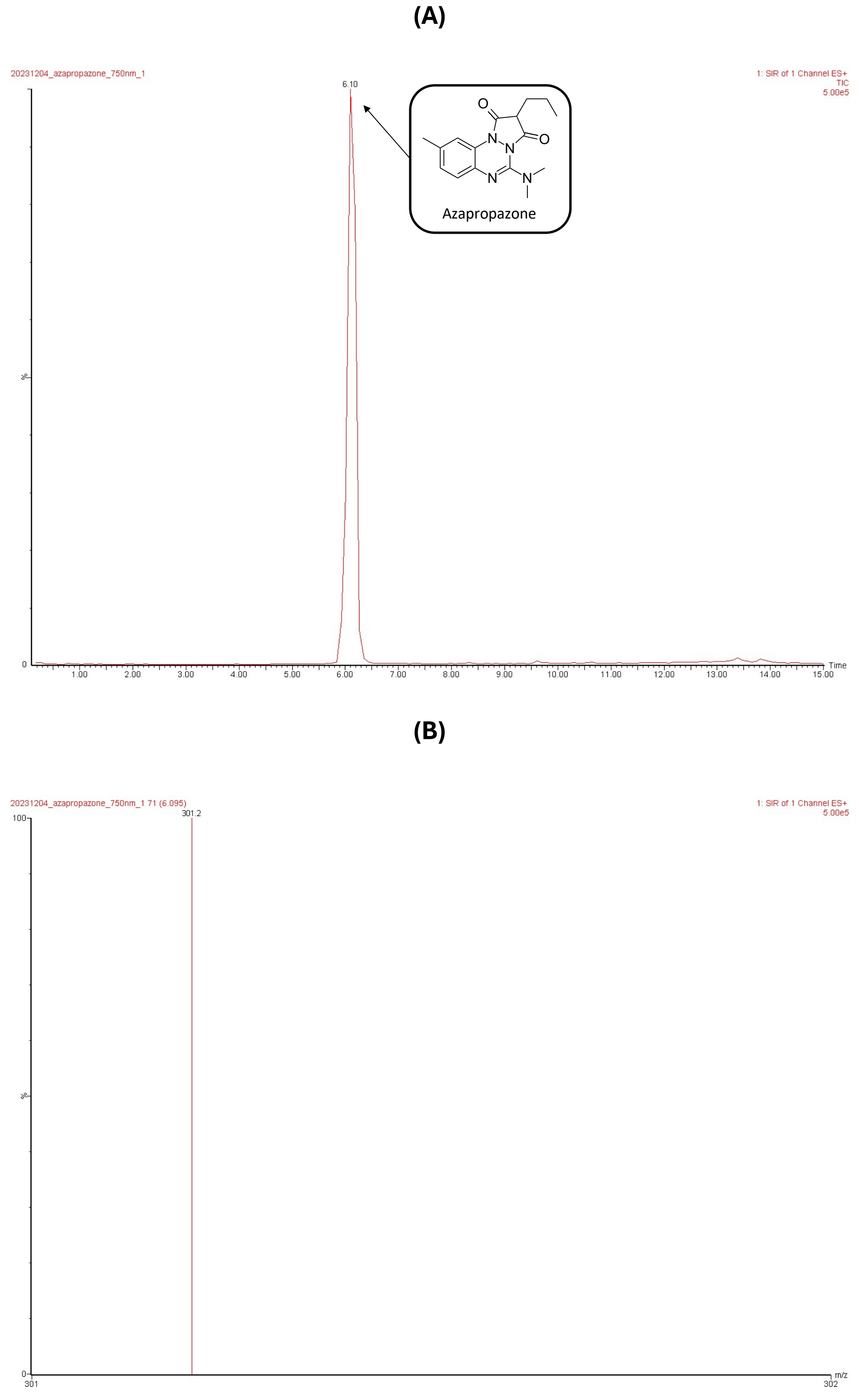

FigS3. Representative LC chromatogram (A) and MS spectra (B) of Azapropazone

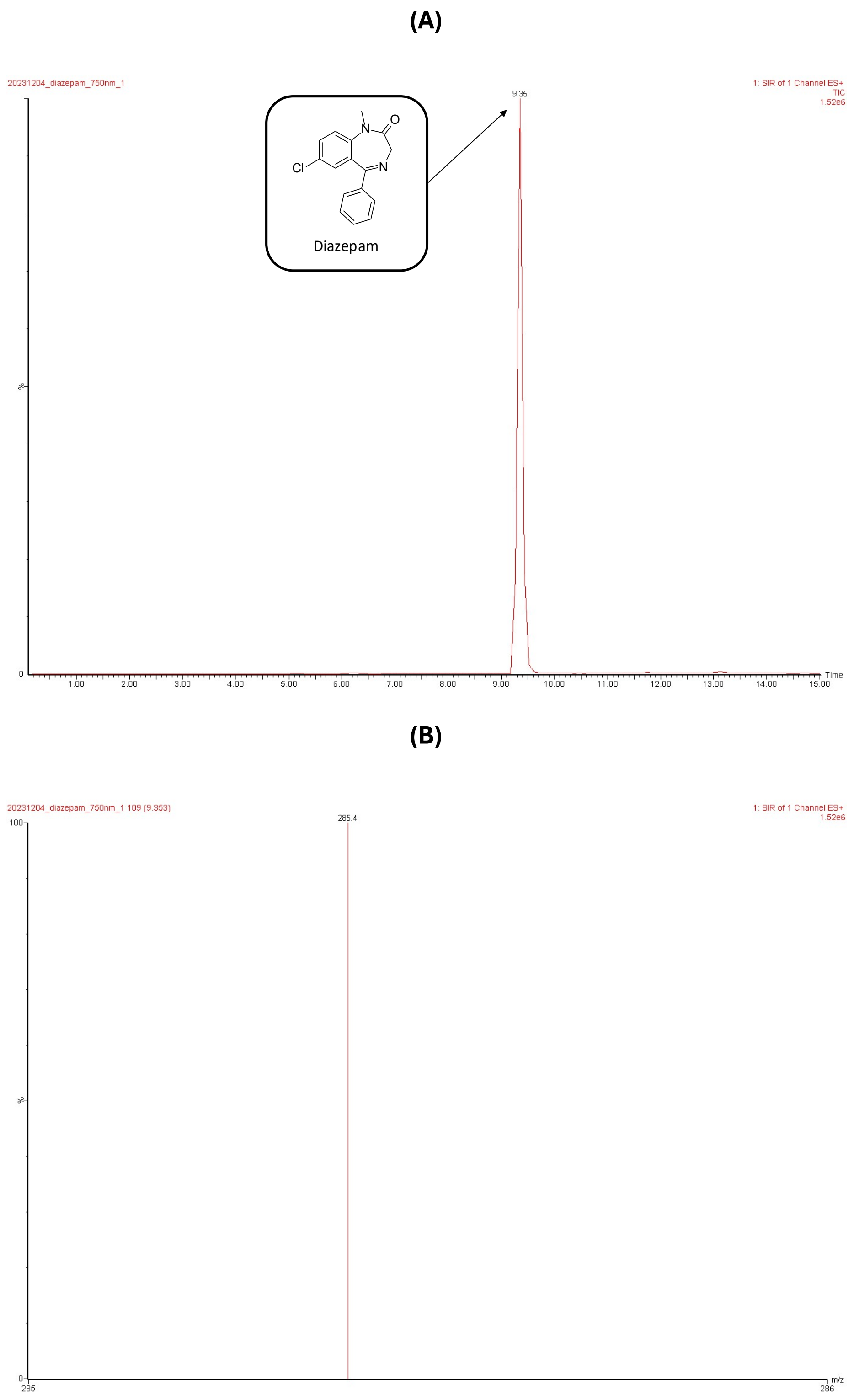

FigS4. Representative LC chromatogram (A) and MS spectra (B) of Diazepam

(A)

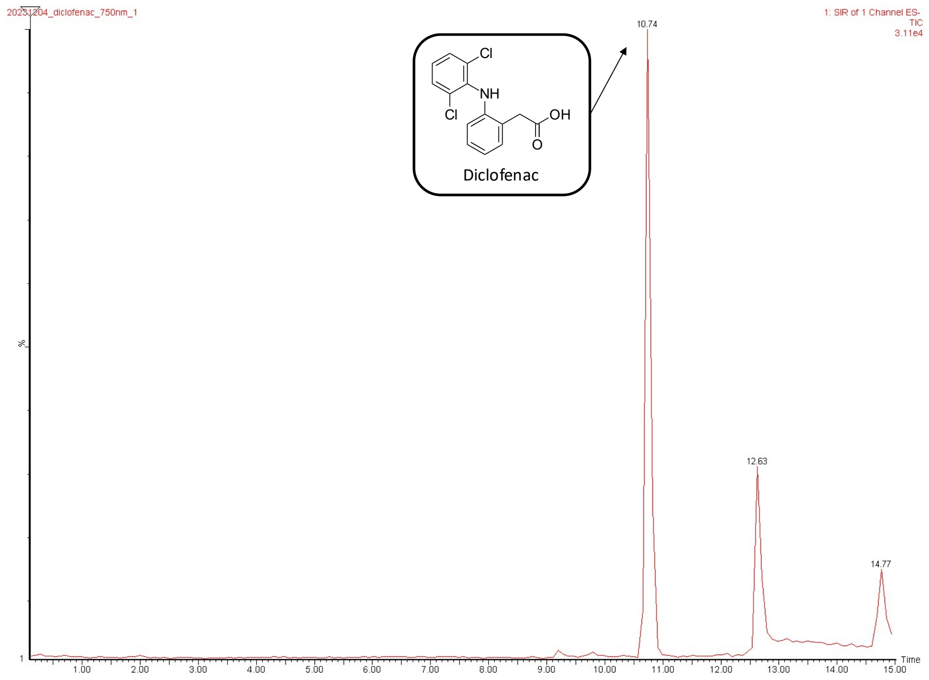

(B)

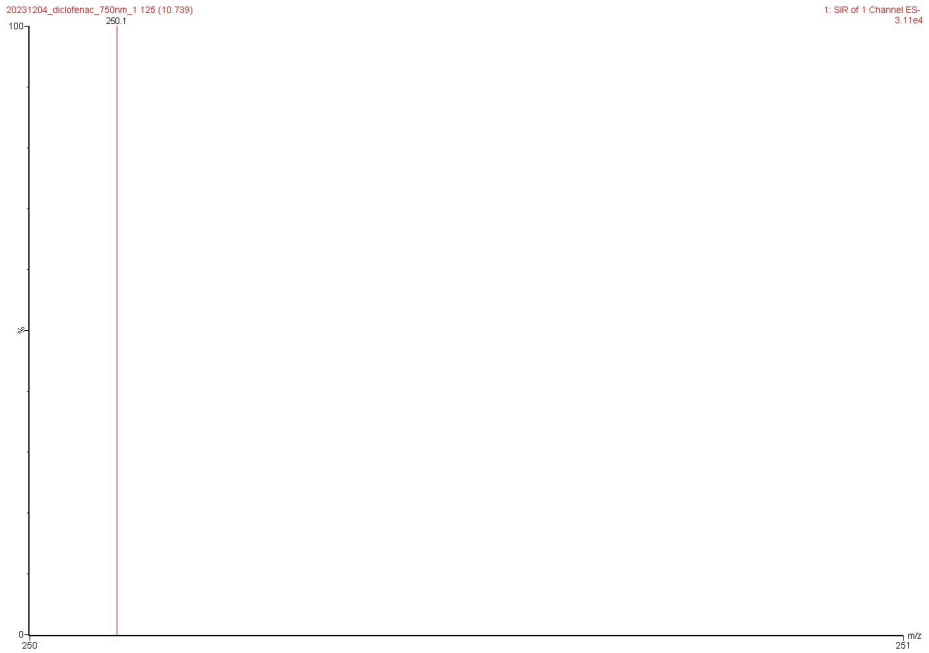

FigS5. Representative LC chromatogram (A) and MS spectra (B) of Diclofenac

(A)

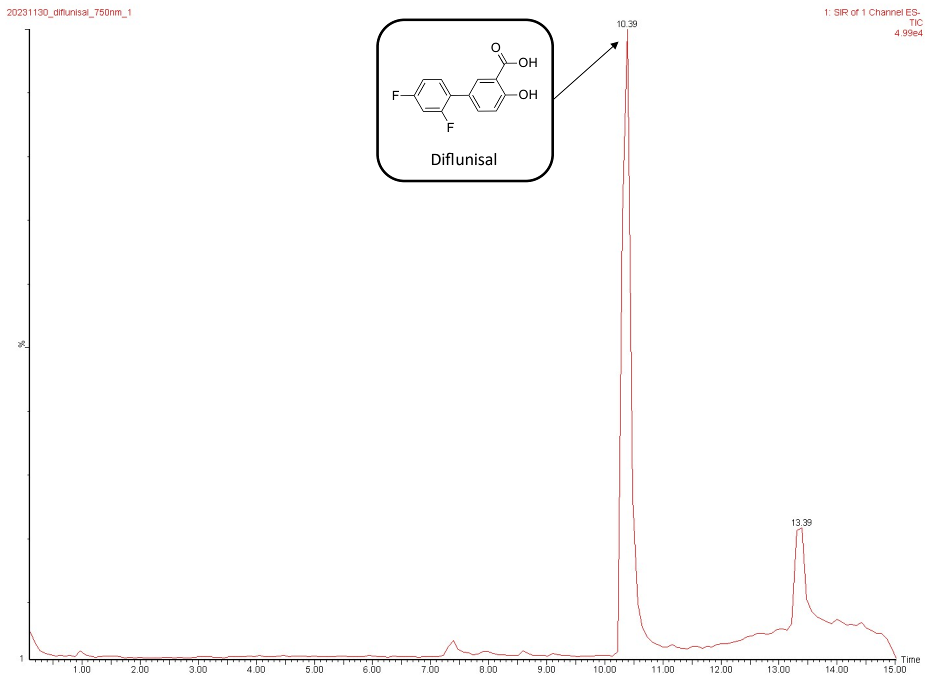

(B)

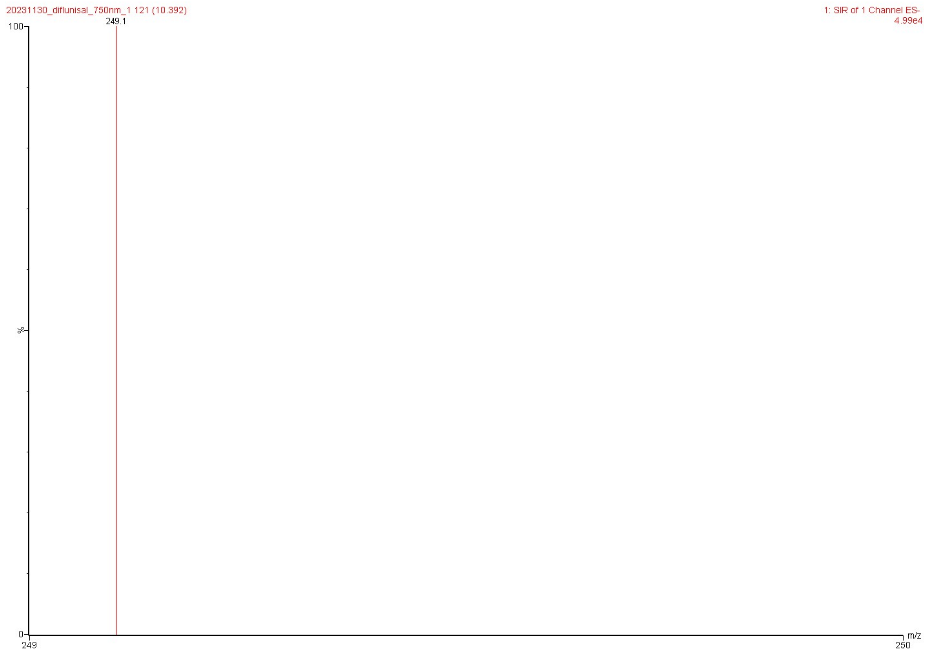

FigS6. Representative LC chromatogram (A) and MS spectra (B) of Diflunisal

(A)

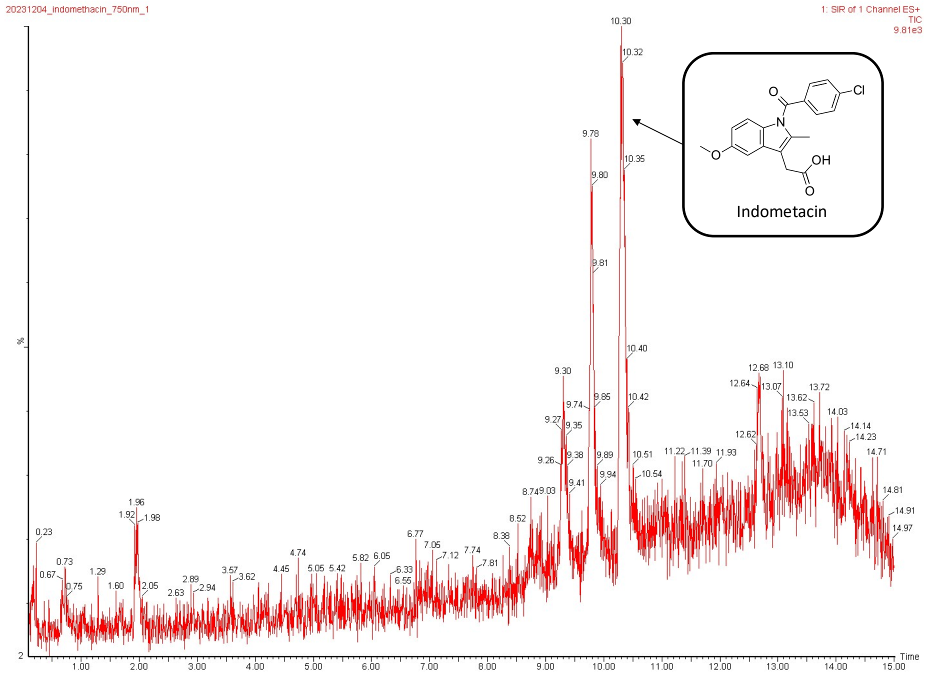

(B)

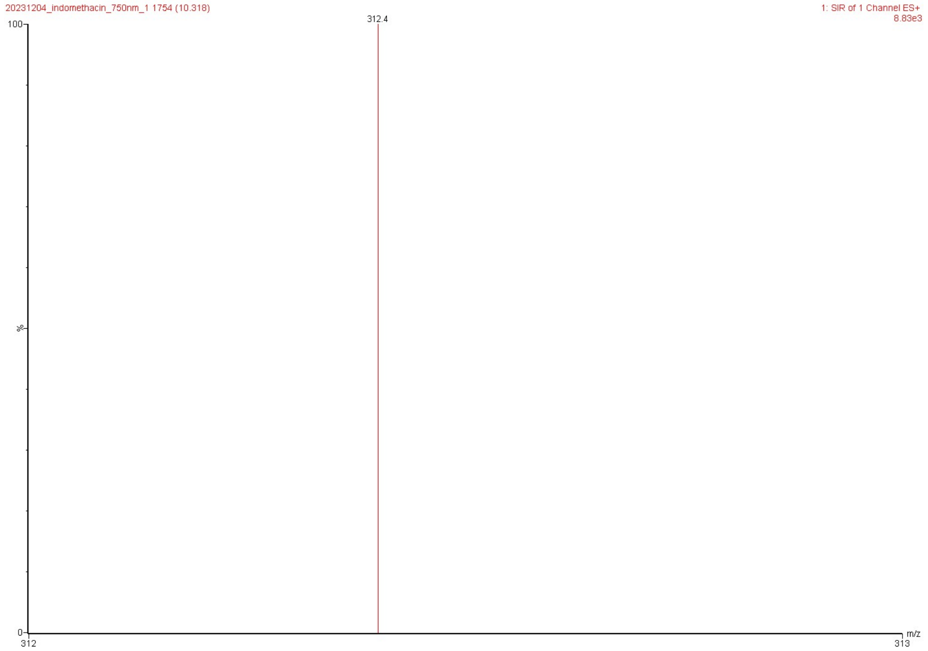

FigS7. Representetive LC chromatogram (A) and MS spectra (B) of Indometacin

(A)

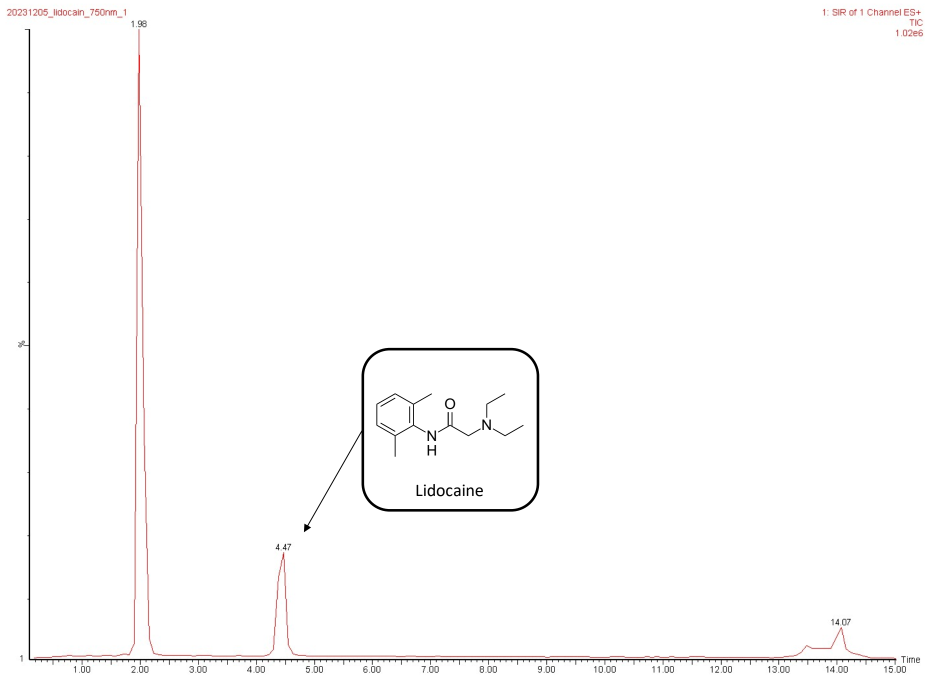

(B)

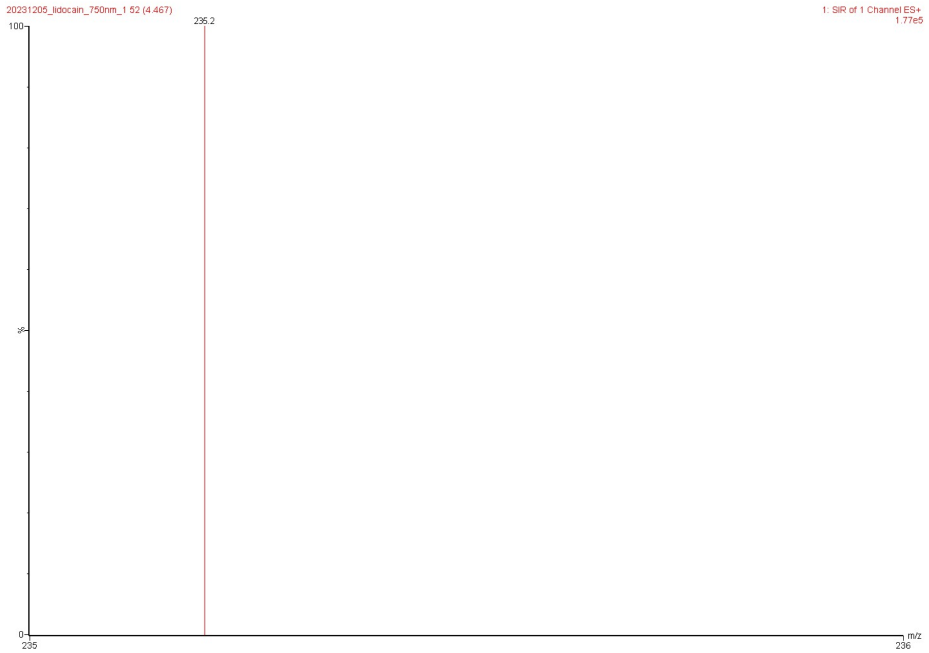

FigS8. Representative LC chromatogram (A) and MS spectra (B) of Lidocaine

(A)

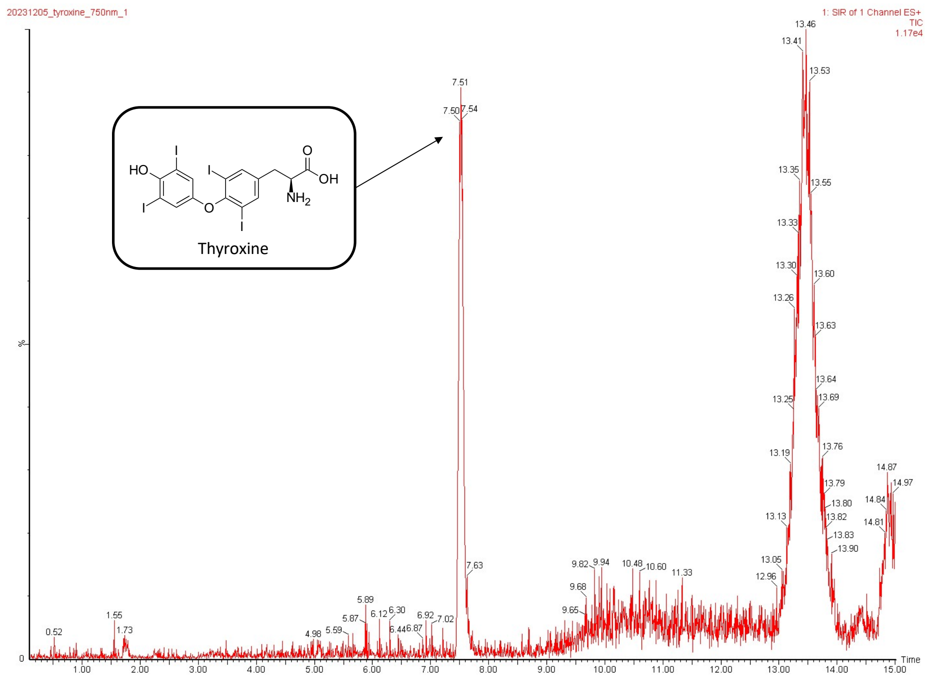

(B)

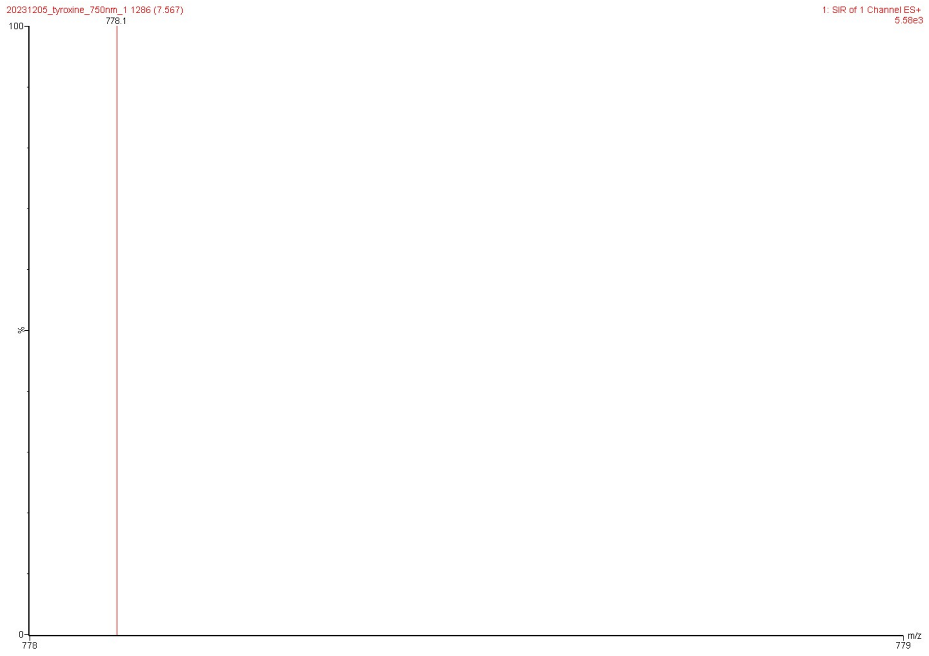

FigS9. Representative LC chromatogram (A) and MS spectra (B) of Thyroxine

(A)

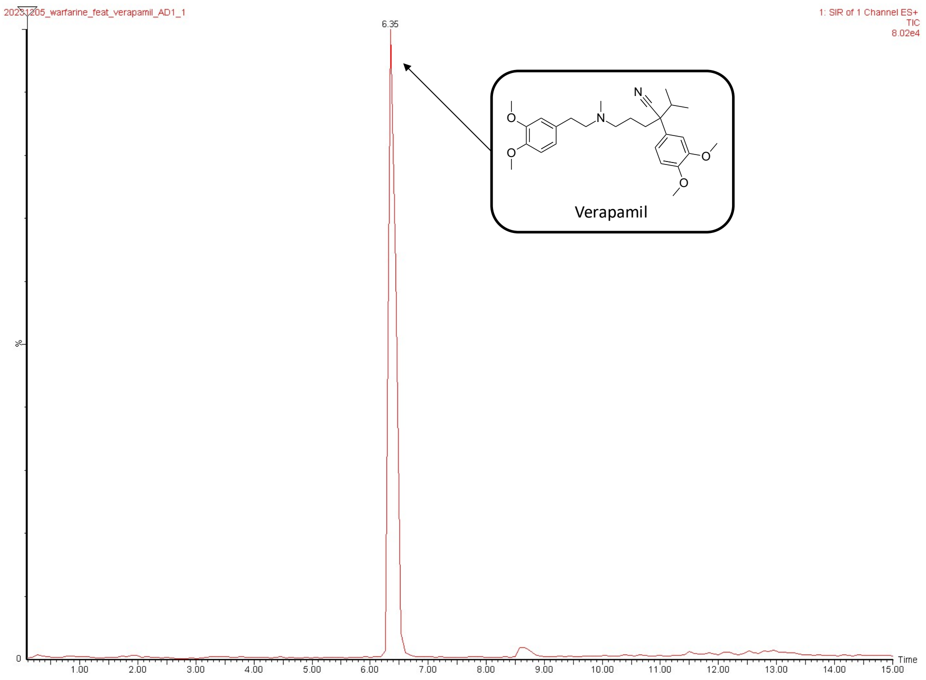

(B)

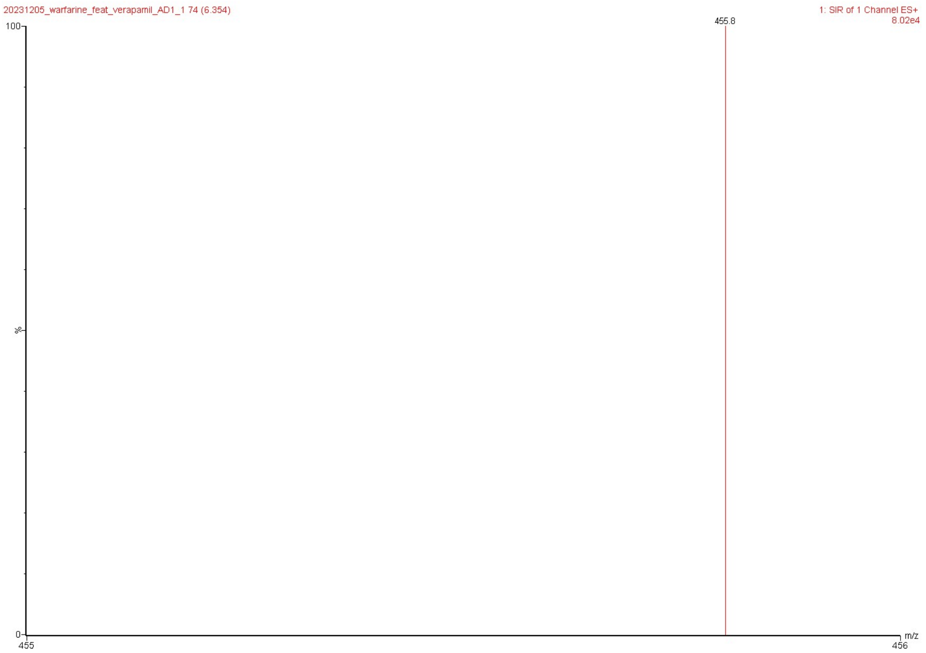

FigS10. Representative LC chromatogram (A) and MS spectra (B) of Verapamil

(A)

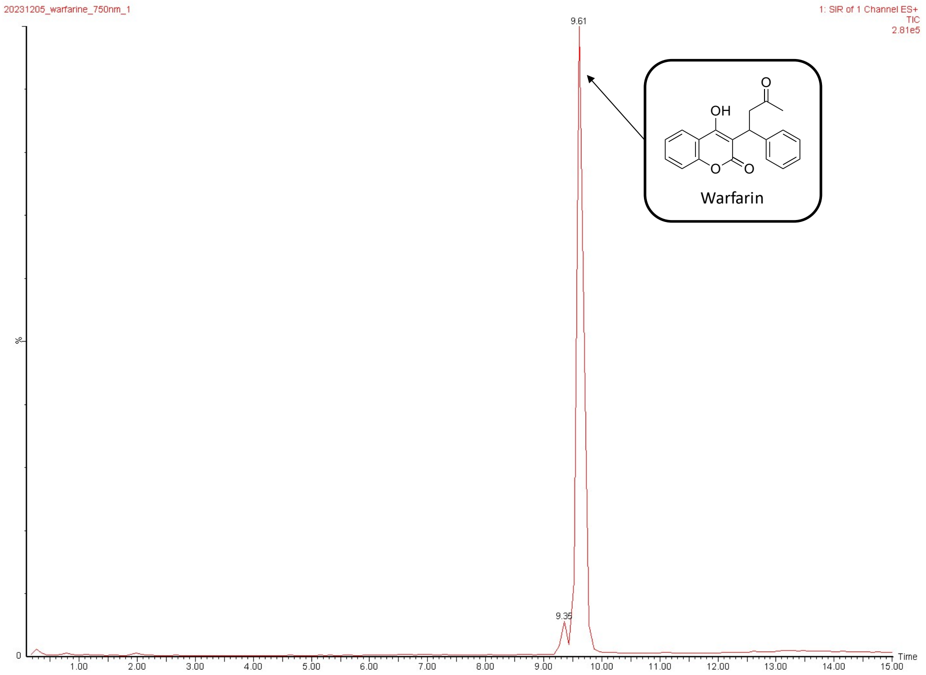

(B)

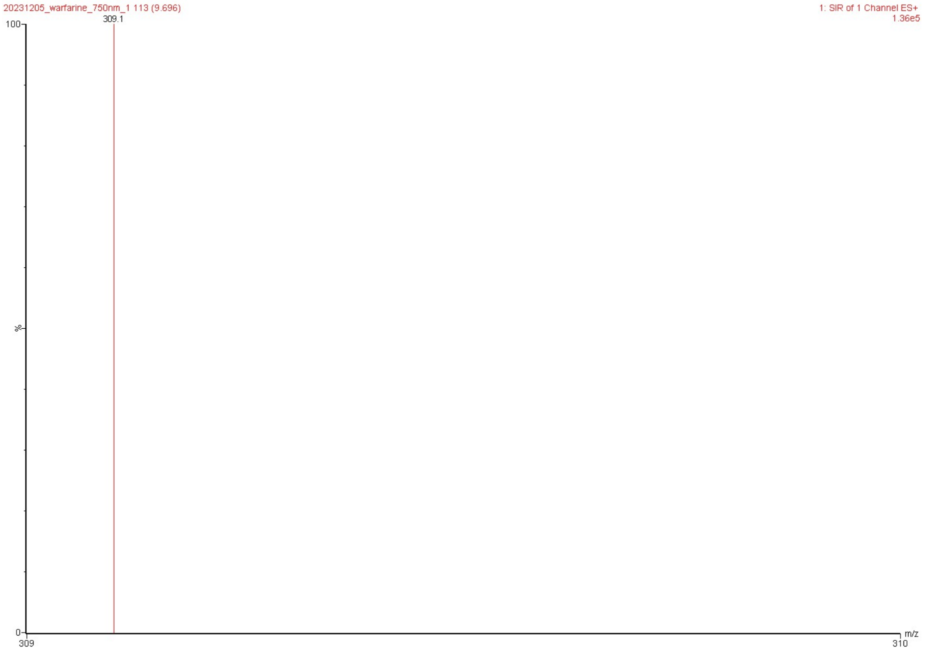

FigS11. Representative LC chromatogram (A) and MS spectra (B) of Warfarin

8. Investigation of enzymatic activity of HSA

8.1 Model substrate, aspirin (ASA)

To investigate the pseudo-esterase activity of HSAs, each HSA solution and acetylsalicylic acid (aspirin, ASA) solution were mixed in the final concentration of 1 mg mL^{-1} and 1 mM in potassium-phosphate buffer (100 mM, pH 7.4) on a 96-well plate (UV-Star® 96 W microplate, Greiner Bio One GmbH, Kremsmünster, Austria). First all HSA was preincubated for 5 sec at 37°C, then the substrate solution was added. The specific enzyme activity was calculated by determine the formation of salicylic acid at λ = 296 nm at 37.0 °C after 30 min by utilizing a Multiskan Sky 96-well plate reader instrument by Thermo Fisher Scientific. The decay of the aspirin concentration by it's chemical hydrolysis was subtracted versus time. In case of immobilized HSA in a 1.5 mL Eppendorf tubes 1 mL aspirin solution (1 mM, potassium-phosphate buffer) was added to the corresponding MNP-HSA complexes (5.0 mg) and was shaken for 60 min at 1100 rpm and 37°C applying a thermomixer (Eppendorf, ThermoScientific). Samples (200 µL, each) were taken from the reaction mixtures after the elapsed time and the absorbance of the samples was measured at λ = 296 nm. The assay was performed according to one of the most used protocols. [https://doi.org/10.1016/j.bcp.2009.10.007]

8.2. Model substrtae, *p*-nitrophenyl acetate (PNPA)

To investigate the esterase activity of HSA, each HSA solution and *p*-nitrophenyl acetate (PNPA) solution were mixed in the final concentration of 3.3 mg mL^{-1} and 0.1 mM in potassium-phosphate buffer (100 mM, pH 7.4) on a 96-well plate (UV-Star® 96 W microplate, Greiner Bio One GmbH, Kremsmünster, Austria). First all HSA was preincubated for 5 sec at 25°C, then the substrate solution was added.The specific enzyme activity was calculated on the linear ascending phase of the kinetic curve between 0-60 sec by detecting the formation of p-nitrophenol (PNP) at λ = 400 nm, 25.0 °C for 10 min by utilizing a Multiskan Sky 96-well plate reader instrument by Thermo Fisher Scientific. The decay of the PNPA concentration by chemical hydrolisis of the substrate was subtracted versus time. In case of immobilized HSA in a 1.5 mL Eppendorf tubes 1 mL PNPA solution (0.1 mM, potassium-phosphate buffer, 10 v/v ethanol) was added to the corresponding MNP-HSA complexes (5.0 mg) and was shaken for 60 min at 1100 rpm and 25°C applying a thermomixer (Eppendorf, ThermoScientific). Samples (200 µL, each) were taken from the reaction mixtures after the elapsed time and the absorbance of the samples was measured at λ = 400 nm. The assay was performed according to one of the most used protocols [https://doi.org/10.1248/bpb.b16-00011].

9. Results of the literature search about HSA applied for API binding.

List of the publications about the HSA-API binding studies in the time frame 2018–2024, using Scopus scientific reach searching engine.

Not given

1. Bhunia, A., Vojtíšek, P., & Manna, S. C. (2019). DFT/TD-DFT calculation, photophysical properties, DNA/protein binding and catecholase activity of chelating ligand based trigonal bipyramidal copper(II) complexes. Journal of Molecular Structure, 1179, 558–567. https://doi.org/10.1016/j.molstruc.2018.11.021
2. Singh, I., Rani, R., Luxami, V., & Paul, K. (2019). Synthesis of 5-(4-(1H-phenanthro[9,10-d]imidazol-2-yl)benzylidene)thiazolidine-2,4-dione as promising DNA and serum albumin-binding agents and evaluation of antitumor activity. European Journal of Medicinal Chemistry, 166, 267–280. https://doi.org/10.1016/j.ejmech.2019.01.053
3. Parveen, M., Aslam, A., Alam, M., Siddiqui, M. F., Bano, B., Azaz, S., Silva, M. R., & Silva, P. S. P. (2019). Synthesisand Characterization of Benzothiophene-3-carbonitrile Derivative and Its Interactions with Human Serum Albumin (HSA). ChemistrySelect, 4(41), 11979–11986. https://doi.org/10.1002/slct.201902378
4. Correa, R. S., Oliveira, K. M., Pérez, H., Plutín, A. M., Ramos, R., Mocelo, R., Castellano, E. E., & Batista, A. A. (2019). cis-bis(N-benzoyl-N',N'-dibenzylthioureido)platinum(II): Synthesis, molecular structure and its interaction with human and bovine serum albumin. Arabian Journal of Chemistry, 12(8), 3454–3462. https://doi.org/10.1016/j.arabjc.2015.10.006
5. Shokrollahi, S., Amiri, A., Fadaei-Tirani, F., & Schenk-Joß, K. (2020). Promising anti-cancer potency of 4,5,6,7-tetrahydrobenzo[d]thiazole-based Schiff-bases. Journal of Molecular Liquids, 300, 112262. https://doi.org/10.1016/j.molliq.2019.112262
6. Neto, J. S. S., Krüger, R., Balaguez, R. A., Fronza, M. G., Acunha, T. v., Oliboni, R. S., Savegnago, L., Iglesias, B. A., & Alves, D. (2020). Synthesis, photophysics and biomolecule interactive studies of new hybrid benzo-2,1,3-thiadiazoles. New Journal of Chemistry, 44(7), 2768–2780. https://doi.org/10.1039/C9NJ05932F
7. Yinhua, D., Foroughi, M. M., Aramesh-Boroujeni, Z., Jahani, S., Peydayesh, M., Borhani, F., Khatami, M., Rohani, M., Dusek, M., & Eigner, V. (2020). The synthesis, characterization, DNA/BSA/HSA interactions, molecular modeling, antibacterial properties, and in vitro cytotoxic activities of novel parent and niosome nano-encapsulated Ho(<scp>iii</scp>) complexes. RSC Advances, 10(39), 22891–22908. https://doi.org/10.1039/D0RA03436C
8. Khan, I. M., Shakya, S., Akhtar, R., Alam, K., Islam, M., & Alam, N. (2020). Exploring interaction dynamics of designed organic cocrystal charge transfer complex of 2-hydroxypyridine and oxalic acid with human serum albumin: Single crystal, spectrophotometric, theoretical and antimicrobial studies. Bioorganic Chemistry, 100, 103872. https://doi.org/10.1016/j.bioorg.2020.103872
9. Nolte, W. M., Tessman, R. T., & Goldman, J. L. (2020). Screening trimethoprim primary metabolites for covalent binding to albumin. Medicinal Chemistry Research, 29(7), 1238–1246. https://doi.org/10.1007/s00044-020-02570-z
10. Das, M., Mukherjee, S., Koley, B., Choudhuri, I., Bhattacharyya, N., Roy, P., Samanta, B. C., Barai, M., & Maity, T. (2020). Developing novel zinc(<scp>ii</scp>) and copper(<scp>ii</scp>) Schiff base complexes: combined experimental and theoretical investigation on their DNA/protein binding efficacy and anticancer activity. New Journal of Chemistry, 44(42), 18347–18361. https://doi.org/10.1039/D0NJ03844J
11. Khan, A., Paul, K., Singh, I., Jasinski, J. P., Smolenski, V. A., Hotchkiss, E. P., Kelley, P. T., Shalit, Z. A., Kaur, M., Banerjee, S., Roy, P., & Sharma, R. (2020). Copper(<scp>i</scp>) and silver(<scp>i</scp>) complexes of anthraldehyde thiosemicarbazone: synthesis, structure elucidation,

in vitro anti-tuberculosis/cytotoxic activity and interactions with DNA/HSA. Dalton Transactions, 49(47), 17350–17367. https://doi.org/10.1039/D0DT03104F

12. Mayer, J. C. P., Acunha, T. v., Rodrigues, O. E. D., Back, D. F., Chaves, O. A., Dornelles, L., & Iglesias, B. A. (2021). Synthesis, spectroscopic characterization and DNA/HSA binding studies of (phenyl/naphthyl)ethenyl-substituted 1,3,4-oxadiazolyl-1,2,4-oxadiazoles. New Journal of Chemistry, 45(1), 471–484. https://doi.org/10.1039/D0NJ04530F
13. Stefanello, F. S., Kappenberg, Y. G., Ketzer, A., Franceschini, S. Z., Salbego, P. R. S., Acunha, T. v., Nogara, P. A., Rocha, J. B. T., Martins, M. A. P., Zanatta, N., Iglesias, B. A., & Bonacorso, H. G. (2021). New 1-(Spiro[chroman-2,1'-cycloalkan]-4-yl)-1H-1,2,3-Triazoles: Synthesis, QTAIM/MEP analyses, and DNA/HSA-binding assays. Journal of Molecular Liquids, 324, 114729. https://doi.org/10.1016/j.molliq.2020.114729
14. Parsekar, S. U., Haldar, P., Antharjanam, P. K. S., Kumar, M., & Koley, A. P. (2021). Synthesis, characterization, crystal structure, DNA and human serum albumin interactions, as well as antiproliferative activity of a Cu(II) complex containing a Schiff base ligand formed in situ from the Cu(II)-induced cyclization of 1,5-bis(salicylidene)thiocarbohydrazide. Applied Organometallic Chemistry, 35(4). https://doi.org/10.1002/aoc.6152
15. Paliwal, K., Haldar, P., Antharjanam, P. K. S., & Kumar, M. (2023). Synthesis, Characterization, DNA/HSA Interaction, and Cytotoxic Activity of a Copper(II) Thiolate Schiff Base Complex and Its Corresponding Water-Soluble Stable Sulfinato–O Complex Containing Imidazole as a Co-ligand. ACS Omega, 8(24), 21948–21968. https://doi.org/10.1021/acsomega.3c01853
16. Bonpandi, E., & Kandasamy, N. (2023). Design, Synthesis, Characterization, Anti-Microbial, Anti-Oxidant, DNA, HSA, Cytotoxicity and Anti-Inflammatory Studies of Nitrogen-Substituted Chrysin Derivatives and Metal(II) Complexes. Journal of Cluster Science, 34(4), 2113–2134. https://doi.org/10.1007/s10876-022-02372-w
17. Jevtovic, V., Alhar, M. S. O., Milenković, D., Marković, Z., Dimitrić Marković, J., & Dimić, D. (2023). Synthesis, Structural Characterization, Cytotoxicity, and Protein/DNA Binding Properties of Pyridoxylidene-Aminoguanidine-Metal (Fe, Co, Zn, Cu) Complexes. International Journal of Molecular Sciences, 24(19), 14745. https://doi.org/10.3390/ijms241914745
18. Gupta, S., & Paul, K. (2023). Membrane-active substituted triazines as antibacterial agents against Staphylococcus aureus with potential for low drug resistance and broad activity. European Journal of Medicinal Chemistry, 258, 115551. https://doi.org/10.1016/j.ejmech.2023.115551
19. Teixeira, R. G., Mészáros, J. P., Matos, B., Côrte-Real, L., Xavier, C. P. R., Fontrodona, X., Garcia, M. H., Romero, I., Spengler, G., Vasconcelos, M. H., Tomaz, A. I., Enyedy, É. A., & Valente, A. (2023). Novel family of [RuCp(N,N)(P)]+ compounds with simultaneous anticancer and antibacterial activity: Biological evaluation and solution chemistry studies. European Journal of Medicinal Chemistry, 262, 115922. https://doi.org/10.1016/j.ejmech.2023.115922
20. Ain, Q. U., Singh, A., Singh, I., Carmieli, R., & Sharma, R. (2023). Synthesis, characterization and anti-tubercular activities of copper(II) complexes of substituted 2,3-isatin bisthiosemicarbazones: An experimental and theoretical approach. Results in Chemistry, 6, 101171. https://doi.org/10.1016/j.rechem.2023.101171

Shanghai, Yuenye

SR12018:

1. Cheng, C., Zhou, J., Liao, J., Li, Y., Wang, L., Liu, H., & Wu, L. (2024). Investigation on the interactions of contaminant triclosan with human serum albumin: Spectroscopic and molecular docking studies. Journal of Molecular Structure, 1295, 136737. https://doi.org/10.1016/j.molstruc.2023.136737
2. Zhou, J., Cheng, C., Ma, L., Wu, Y., Zhang, Y., Li, L., Yang, A., & Wu, L. (2023). Investigating the interactions of benzoylaconine and benzoylhypacoitine with human serum albumin: Experimental studies and computer calculations. Journal of Molecular Structure, 1294, 136497. https://doi.org/10.1016/j.molstruc.2023.136497

Unknown type:

1. Guo, Q., Liu, M., Zhao, Y., Wu, Y., Liu, J., Cai, C., Shi, Y., & Han, J. (2019). Spectroscopic and cytotoxicity studies on the combined interaction of (−)-epigallocatechin-3-gallate and anthracycline drugs with human serum albumin. Spectrochimica Acta Part A: Molecular and Biomolecular Spectroscopy, 222, 117213. https://doi.org/10.1016/j.saa.2019.117213
2. Liu, T., Liu, M., Guo, Q., Liu, Y., Zhao, Y., Wu, Y., Sun, B., Wang, Q., Liu, J., & Han, J. (2020). Investigation of binary and ternary systems of human serum albumin with oxyresveratrol/piceatannol and/or mitoxantrone by multipectroscopy, molecular docking and cytotoxicity evaluation. Journal of Molecular Liquids, 311, 113364. https://doi.org/10.1016/j.molliq.2020.113364
3. Zhou, B., Zhou, H., Xu, L., Cai, R., Chen, C., Chi, B., & Tuo, X. (2022). An insight into the interaction between Indisulam and human serum albumin: Spectroscopic method, computer simulation and in vitro cytotoxicity assay. Bioorganic Chemistry, 127, 106017. https://doi.org/10.1016/j.bioorg.2022.106017
4. Zhu, J., Liu, S., Dai, L., Yu, F., Zhou, T., Chen, J., Xu, J., Yu, B., Tang, S., Liu, Q., Yang, X.-L., & Han, X.-L. (2024). Elucidating the interaction between equisetin and human serum albumin: A comprehensive study using spectroscopy, microcalorimetry and molecular docking approaches. Spectrochimica Acta Part A: Molecular and Biomolecular Spectroscopy, 304, 123409. https://doi.org/10.1016/j.saa.2023.123409

Solarbio:

1. Zhang, Q., Zhu, Z., & Ni, Y. (2020). Interaction between aspirin and vitamin C with human serum albumin as binary and ternary systems. Spectrochimica Acta Part A: Molecular and Biomolecular Spectroscopy, 236, 118356. https://doi.org/10.1016/j.saa.2020.118356
2. Han, X., Sun, J., Niu, T., Mao, B., Gao, S., Zhao, P., & Sun, L. (2022). Molecular Insight into the Binding of Astilbin with Human Serum Albumin and Its Effect on Antioxidant Characteristics of Astilbin. Molecules, 27(14), 4487. https://doi.org/10.3390/molecules27144487
3. Niu, T., Zhu, X., Zhao, D., Li, H., Yan, P., Zhao, L., Zhang, W., Zhao, P., & Mao, B. (2023). Unveiling interaction mechanisms between myricitrin and human serum albumin: Insights from multi-spectroscopic, molecular docking and molecular dynamic simulation analyses. Spectrochimica Acta. Part A, Molecular and Biomolecular Spectroscopy, 285, 121871. https://doi.org/10.1016/j.saa.2022.121871
4. Li, X., Yan, X., Yang, D., Chen, S., & Yuan, H. (2023). Probing the Interaction between Isoflucypram Fungicides and Human Serum Albumin: Multiple Spectroscopic and Molecular Modeling Investigations. International Journal of Molecular Sciences, 24(15), 12521. https://doi.org/10.3390/ijms241512521

Other:

Himedia:

1. Vidhyapriya, P., Divya, D., Manimaran, Bala., & Sakthivel, N. (2019). Molecular interaction of manganese based carbon monoxide releasing molecule (MnCORM) with human serum albumin (HSA). Bioorganic Chemistry, 92, 103078. https://doi.org/10.1016/j.bioorg.2019.103078

TCI Chemicals:

1. Sarkar, P., Gupta, S., Udaya Kumar, A. H., Das, D., Sutradhar, S., Paul, K., Lokanath, N. K., & Nath Ghosh, B. (2023). Protein interactions and drug displacement studies of novel copper(II) and zinc(II) complexes of a dipyrazinylpyridine ligand. Journal of Molecular Liquids, 387, 122561. https://doi.org/10.1016/j.molliq.2023.122561

Fermentas Life Sciences:

1. Jayasri, B., Rajeshwari, K., Vasantha, P., & Anantha Lakshmi, P. v. (2023). Ternary Cobalt (II)-Metformin-Glycine/Histidine/Proline Complexes: Multispectroscopic DNA, HSA, and BSA Interaction and Cytotoxicity Studies. Biological Trace Element Research, 201(11), 5481–5499. https://doi.org/10.1007/s12011-023-03606-2

Sigma-Aldrich / Merck:

Not specified:

With purity data:

1. Shahraki, S., Majd, M. H., & Heydari, A. (2019). Novel tetradentate Schiff base zinc(II) complex as a potential antioxidant and cancer chemotherapeutic agent: Insights from the photophysical and computational approach. Journal of Molecular Structure, 1177, 536–544. https://doi.org/10.1016/j.molstruc.2018.10.005
2. Żołek, T., Dömötör, O., Ostrowska, K., Enyedy, É. A., & Maciejewska, D. (2019). Evaluation of blood-brain barrier penetration and examination of binding to human serum albumin of 7-O-arylpiperazinylcoumarins as potential antipsychotic agents. Bioorganic Chemistry, 84, 211–225. https://doi.org/10.1016/j.bioorg.2018.11.034
3. Naik, R., & Jaldappagari, S. (2019). Spectral and computational attributes: Binding of a potent anticancer agent, dasatinib to a transport protein. Journal of Molecular Liquids, 293, 111492. https://doi.org/10.1016/j.molliq.2019.111492
4. Shaghaghi, M., Dehghan, G., Rashtbari, S., Sheibani, N., & Aghamohammadi, A. (2019). Multispectral and computational probing of the interactions between sitagliptin and serum albumin. Spectrochimica Acta Part A: Molecular and Biomolecular Spectroscopy, 223, 117286. https://doi.org/10.1016/j.saa.2019.117286
5. Sindhu, A., Bhakuni, K., Sankaranarayanan, K., & Venkatesu, P. (2020). Implications of Imidazolium-Based Ionic Liquids as Refolding Additives for Urea-Induced Denatured Serum Albumins. ACS Sustainable Chemistry & Engineering, 8(1), 604–612. https://doi.org/10.1021/acssuschemeng.9b06194
6. Zhang, J., Gao, X., Huang, J., & Wang, H. (2020). Probing the Interaction between Human Serum Albumin and 9-Hydroxyphenanthrene: A Spectroscopic and Molecular Docking Study. ACS Omega, 5(27), 16833–16840. https://doi.org/10.1021/acsomega.0c02031
7. Zhang, H., Deng, H., & Wang, Y. (2020). Comprehensive investigations about the binding interaction of acesulfame with human serum albumin. Spectrochimica Acta Part A: Molecular and Biomolecular Spectroscopy, 237, 118410. https://doi.org/10.1016/j.saa.2020.118410
8. Xin, X., Chen, L., Li, Y., Yu, R., Fan, H., Yan, Z., Li, S., & Feng, H. (2021). Study on the interaction of hyperoside and human serum albumin in V C and V C -free environments by spectroscopic and molecular docking techniques. Luminescence, 36(3), 595–605. https://doi.org/10.1002/bio.3978
9. Shamsi, A., Shahwan, M., Khan, M. S., Alhumaydhi, F. A., Alsagaby, S. A., al Abdulmonem, W., Abdullaev, B., & Yadav, D. K. (2022). Mechanistic Insight into Binding of Huperzine A with Human Serum Albumin: Computational and Spectroscopic Approaches. Molecules, 27(3), 797. https://doi.org/10.3390/molecules27030797
10. Banu, A., Khan, R. H., Qashqoosh, M. T. A., Manea, Y. K., Furkan, M., & Naqvi, S. (2022). Multispectroscopic and computational studies of interaction of bovine serum albumin, human serum albumin and bovine hemoglobin with bisacodyl. Journal of Molecular Structure, 1249, 131550. https://doi.org/10.1016/j.molstruc.2021.131550
11. Li, S., Tonelli, M., & Unsworth, L. D. (2022). Indoxyl and p-cresol sulfate binding with human serum albumin. Colloids and Surfaces A: Physicochemical and Engineering Aspects, 635, 128042. https://doi.org/10.1016/j.colsurfa.2021.128042
12. Jagusiak, A., Chłopaś, K., Zemanek, G., Kościk, I., Skorek, P., & Stopa, B. (2022). Albumin Binds Doxorubicin via Self–Assembling Dyes as Specific Polymolecular Ligands. International Journal of Molecular Sciences, 23(9), 5033. https://doi.org/10.3390/ijms23095033

13. Masternak, J., Gilewska, A., Kowalik, M., Kazimierczuk, K., Sitkowski, J., Okła, K., Wietrzyk, J., & Barszcz, B. (2022). Synthesis, crystal structure and spectroscopic characterization of new anionic iridium(III) complexes and their interaction with biological targets. Polyhedron, 221, 115837. https://doi.org/10.1016/j.poly.2022.115837
14. Li, M., Zhang, J., Zhu, Y., & Zhang, Y. (2022). Interactions between hydroxylated polycyclic aromatic hydrocarbons and serum albumins: multispectral and molecular docking analyses. Luminescence, 37(11), 1972–1981. https://doi.org/10.1002/bio.4384
15. Saraswat, J., Kumar, S., Alzahrani, K. A., Malik, M. A., & Patel, R. (2023). Experimental and Computational Characterisation of the Molecular Interactions between 1-Butyl-1-methyl-pyrrolidin-1-ium bis(trifluoromethanesulphonyl)imide and Human Serum Albumin. ChemistrySelect, 8(1). https://doi.org/10.1002/slct.202204159
16. Amir, M., & Javed, S. (2023). Elucidation of binding dynamics of tyrosine kinase inhibitor tepotinib, to human serum albumin, using spectroscopic and computational approach. International Journal of Biological Macromolecules, 241, 124656. https://doi.org/10.1016/j.ijbiomac.2023.124656
17. Rahman, N., & Khalil, N. (2023). Characterization of sulfasalazine-bovine serum albumin and human serum albumin interaction by spectroscopic and theoretical approach. Spectrochimica Acta Part A: Molecular and Biomolecular Spectroscopy, 300, 122865. https://doi.org/10.1016/j.saa.2023.122865
18. Huang, S., Cao, H., Tu, X., Xie, J., Su, W., & Xiao, Q. (2023). Comparative investigation on interaction mechanism and native conformation of human serum albumin with organometallic iridium(III) complexes via spectroscopic and electrochemical approaches. Journal of Molecular Structure, 1291, 136017. https://doi.org/10.1016/j.molstruc.2023.136017

Without purity data:

1. Shahabadi, N., Amiri, S., & Taherpour, A. (Arman). (2019). Human serum albumin binding studies of a new platinum(IV) complex containing the drug pregabalin: experimental and computational methods. Journal of Coordination Chemistry, 72(4), 600–618. https://doi.org/10.1080/00958972.2019.1568419
2. Chugh, H., Kumar, P., Tomar, V., Kaur, N., Sood, D., & Chandra, R. (2019). Interaction of noscapine with human serum albumin (HSA): A spectroscopic and molecular modelling approach. Journal of Photochemistry and Photobiology A: Chemistry, 372, 168–176. https://doi.org/10.1016/j.jphotochem.2018.12.001
3. Agrawal, R., Thakur, Y., Tripathi, M., Siddiqi, M. K., Khan, R. H., & Pande, R. (2019). Elucidating the binding propensity of naphthyl hydroxamic acid to human serum albumin (HSA): Multi-spectroscopic and molecular modeling approach. Journal of Molecular Structure, 1184, 1–11. https://doi.org/10.1016/j.molstruc.2019.01.067
4. Fliszár-Nyúl, E., Lemli, B., Kunsági-Máté, S., Dellafiora, L., Dall'Asta, C., Cruciani, G., Pethő, G., & Poór, M. (2019). Interaction of Mycotoxin Alternariol with Serum Albumin. International Journal of Molecular Sciences, 20(9), 2352. https://doi.org/10.3390/ijms20092352
5. Sasmal, M., Islam, A. S. M., Bhowmick, R., Maiti, D., Dutta, A., & Ali, M. (2019). Site-Selective Interaction of Human Serum Albumin with 4-Chloro-7-nitro-1,2,3-benzoxadiazole Modified Olanzapine Derivative and Effect of β-Cyclodextrin on Binding: In the Light of Spectroscopy and Molecular Docking. ACS Applied Bio Materials, 2(8), 3551–3561. https://doi.org/10.1021/acsabm.9b00429
6. Paul, A., Mistri, S., Bertolasi, V., & Manna, S. C. (2019). DNA/protein binding and molecular docking studies of two tetranuclear Cu(II) complexes with double-open-cubane core like structure. Inorganica Chimica Acta, 495, 119005. https://doi.org/10.1016/j.ica.2019.119005
7. de Alcântara-Contessoto, N. S., Caruso, Í. P., Bezerra, D. P., Filho, J. M. B., & Cornélio, M. L. (2019). An investigation into the interaction between piplartine (piperlongumine) and human serum albumin. Spectrochimica Acta Part A: Molecular and Biomolecular Spectroscopy, 220, 117084. https://doi.org/10.1016/j.saa.2019.04.076

8. Zianna, A., Geromichalos, G. D., Pekou, A., Hatzidimitriou, A. G., Coutouli-Argyropoulou, E., Lalia-Kantouri, M., Pantazaki, A. A., & Psomas, G. (2019). A palladium(II) complex with the Schiff base 4-chloro-2-(N-ethyliminomethyl)-phenol: Synthesis, structural characterization, and in vitro and in silico biological activity studies. Journal of Inorganic Biochemistry, 199, 110792. https://doi.org/10.1016/j.jinorgbio.2019.110792
9. Ma, R., Guo, D.-X., Li, H.-F., Liu, H.-X., Zhang, Y.-R., Ji, J.-B., Xing, J., & Wang, S.-Q. (2019). Spectroscopic methodologies and molecular docking studies on the interaction of antimalarial drug piperaquine and its metabolites with human serum albumin. Spectrochimica Acta Part A: Molecular and Biomolecular Spectroscopy, 222, 117158. https://doi.org/10.1016/j.saa.2019.117158
10. Chaves, O. A., Menezes, L. B., & Iglesias, B. A. (2019). Multiple spectroscopic and theoretical investigation of meso-tetra-(4-pyridyl)porphyrin-ruthenium(II) complexes in HSA-binding studies. Effect of Zn(II) in protein binding. Journal of Molecular Liquids, 294, 111581. https://doi.org/10.1016/j.molliq.2019.111581
11. Marković, K., Milačič, R., Marković, S., Kladnik, J., Turel, I., & Ščančar, J. (2020). Binding Kinetics of Ruthenium Pyrithione Chemotherapeutic Candidates to Human Serum Proteins Studied by HPLC-ICP-MS. Molecules, 25(7), 1512. https://doi.org/10.3390/molecules25071512
12. Saswati, Mohanty, M., Banerjee, A., Biswal, S., Horn, A., Schenk, G., Brzezinski, K., Sinn, E., Reuter, H., & Dinda, R. (2020). Polynuclear zinc(II) complexes of thiosemicarbazone: Synthesis, X-ray structure and biological evaluation. Journal of Inorganic Biochemistry, 203, 110908. https://doi.org/10.1016/j.jinorgbio.2019.110908
13. Chanphai, P., & Tajmir-Riahi, H. A. (2020). Conjugation of citric acid and gallic acid with serum albumins: Acid binding sites and protein conformation. Journal of Molecular Liquids, 299, 112178. https://doi.org/10.1016/j.molliq.2019.112178
14. Wani, T. A., Bakheit, A. H., Zargar, S., Rizwana, H., & Al-Majed, A. A. (2020). Evaluation of competitive binding interaction of neratinib and tamoxifen to serum albumin in multidrug therapy. Spectrochimica Acta Part A: Molecular and Biomolecular Spectroscopy, 227, 117691. https://doi.org/10.1016/j.saa.2019.117691
15. Chaves, O. A., Acunha, T. v., Iglesias, B. A., Jesus, C. S. H., & Serpa, C. (2020). Effect of peripheral platinum(II) bipyridyl complexes on the interaction of tetra-cationic porphyrins with human serum albumin. Journal of Molecular Liquids, 301, 112466. https://doi.org/10.1016/j.molliq.2020.112466
16. Singh, I., Luxami, V., & Paul, K. (2020). Spectroscopy and molecular docking approach for investigation on the binding of nocodazole to human serum albumin. Spectrochimica Acta Part A: Molecular and Biomolecular Spectroscopy, 235, 118289. https://doi.org/10.1016/j.saa.2020.118289
17. Banerjee, A., Mohanty, M., Lima, S., Samanta, R., Garribba, E., Sasamori, T., & Dinda, R. (2020). Synthesis, structure and characterization of new dithiocarbazate-based mixed ligand oxidovanadium(<scp>iv</scp>) complexes: DNA/HSA interaction, cytotoxic activity and DFT studies. New Journal of Chemistry, 44(26), 10946–10963. https://doi.org/10.1039/D0NJ01246G
18. Chaves, O. A., Fernandes, T. V. A., de Melos, J. L. R., Netto-Ferreira, J. C., & Echevarria, A. (2020). Elucidation of the interaction between human serum albumin (HSA) and 3,4-methylenedioxyde-6-iodo-benzaldehyde-thiosemicarbazone, a potential drug for Leishmania amazonensis: Multiple spectroscopic and dynamics simulation approach. Journal of Molecular Liquids, 310, 113117. https://doi.org/10.1016/j.molliq.2020.113117
19. wang, Y., Liu, Y., Yang, Q., Mao, X., Chai, W.-M., & Peng, Y. (2020). Study on the interaction between 4-(1H-indol-3-yl)-2-(p-tolyl)quinazoline-3-oxide and human serum albumin. Bioorganic & Medicinal Chemistry, 28(21), 115720. https://doi.org/10.1016/j.bmc.2020.115720
20. Martínez, A., Zahran, M., Gomez, M., Guevara, J., Pichardo-Bueno, R., Asim, J., Ortiz, G., Andoh, Y., Shibutani, S., & Kaur, B. (2020). Ionophoric polyphenols are permeable to the blood–brain barrier, interact with human serum albumin and Calf Thymus DNA, and inhibit AChE enzymatic activity. Medicinal Chemistry Research, 29(11), 1956–1975. https://doi.org/10.1007/s00044-020-02615-3

21. Liao, T., Zhang, Y., Huang, X., Jiang, Z., & Tuo, X. (2021). Multi-spectroscopic and molecular docking studies of human serum albumin interactions with sulfametoxydiazine and sulfamonomethoxine. Spectrochimica Acta Part A: Molecular and Biomolecular Spectroscopy, 246, 119000. https://doi.org/10.1016/j.saa.2020.119000
22. Wani, T. A., Bakheit, A. H., Zargar, S., Alanazi, Z. S., & Al-Majed, A. A. (2021). Influence of antioxidant flavonoids quercetin and rutin on the in-vitro binding of neratinib to human serum albumin. Spectrochimica Acta Part A: Molecular and Biomolecular Spectroscopy, 246, 118977. https://doi.org/10.1016/j.saa.2020.118977
23. Barghash, S., El-Razeq, S. A., Elmansi, H., Elmorsy, M. A., & Belal, F. (2022). Intermolecular Interactions of Saxagliptin and Vildagliptin with Human Serum Albumin. Journal of Applied Spectroscopy, 88(6), 1266–1275. https://doi.org/10.1007/s10812-022-01308-6
24. Raghu, M. S., Kumar, K. Y., Veena, K., Kumar, C. B. P., Almalki, A. S., Mani, G., Alasmary, F. A., & Prashanth, M. K. (2022). Synthesis, characterization, antimicrobial and interaction studies of pteridines with human serum albumin: A combined multi-spectroscopic and computational study. Journal of Molecular Structure, 1250, 131857. https://doi.org/10.1016/j.molstruc.2021.131857
25. Cai, D.-H., Chen, B.-H., Liu, Q.-Y., Le, X.-Y., & He, L. (2022). Synthesis, structural studies, interaction with DNA/HSA and antitumor evaluation of new Cu(<scp>ii</scp>) complexes containing 2-(1 H -imidazol-2-yl)pyridine and amino acids. Dalton Transactions, 51(43), 16574–16586. https://doi.org/10.1039/D2DT02985E
26. Huang, Z.-Y., Li, X.-Y., Hu, L.-Y., Bai, A.-M., & Hu, Y.-J. (2022). Comparative study of two antipsychotic drugs binding to human serum albumin: By multispectroscopic and molecular docking methods. Journal of Molecular Liquids, 365, 120084. https://doi.org/10.1016/j.molliq.2022.120084
27. Alshaikh, N. E., Zaki, M., Sharfalddin, A. A., Al-Radadi, N. S., Hussien, M. A., & Hassan, W. M. I. (2023). Synthesis, structural characterization, DNA/HSA binding, molecular docking and anticancer studies of some D-Luciferin complexes. Arabian Journal of Chemistry, 16(7), 104845. https://doi.org/10.1016/j.arabjc.2023.104845
28. Khachatrian, A. A., Mukhametzyanov, T. A., Salikhov, R. Z., Safin, M. v., Yakhvarov, D. G., Garifullin, B. F., Terenteva, O. S., Padnya, P. L., Stoikov, I. I., Voloshina, A. D., & Solomonov, B. N. (2023). The interaction of cholinium-based ionic liquids with different biological origin anions with albumins. Journal of Molecular Liquids, 382, 121995. https://doi.org/10.1016/j.molliq.2023.121995
29. Petrović, Đ. S., Jovičić Milić, S. S., Đukić, M. B., Radojević, I. D., Jurišević, M. M., Gajović, N. M., Petrović, A., Arsenijević, N. N., Jovanović, I. P., Avdović, E., Stojković, D. Lj., & Jevtić, V. v. (2023). Synthesis, characterization, HSA binding, molecular docking, cytotoxicity study, and antimicrobial activity of new palladium(II) complexes with propylenediamine derivatives of phenylalanine. Journal of Inorganic Biochemistry, 246, 112283. https://doi.org/10.1016/j.jinorgbio.2023.112283
30. Shahraki, S., Delarami, H. S., Poorsargol, M., Razmara, Z., & Majd, M. H. (2023). A Comprehensive Study on the Binding of Anti-cancer Drug (Floxuridine) with Human Serum Albumin. Iranian Journal of Science, 47(4), 1155–1167. https://doi.org/10.1007/s40995-023-01502-x
31. Skuredina, A. A., Kopnova, T. Yu., Belogurova, N. G., & Kudryashova, E. v. (2023). Encapsulation of Ciprofloxacin into a Cyclodextrin Polymer Matrix: The Complex Formation with Human Serum Albumin and In Vitro Studies. Chemistry, 5(3), 1942–1960. https://doi.org/10.3390/chemistry5030132
32. Konovalov, B., Đorđević, I. S., Franich, A. A., Šmit, B., Živković, M. D., Djuran, M. I., Janjić, G. v., & Rajković, S. (2023). Dinuclear platinum(II) complexes with 1,5-nphe bridging ligand: Spectroscopic and molecular docking study of the interactions with N-acetylated L-methionylglycine and human serum albumin. Journal of Molecular Structure, 1288, 135810. https://doi.org/10.1016/j.molstruc.2023.135810
33. Shakibapour, N., Asoodeh, A., Saberi, M. R., & Chamani, J. (2023). Investigating the binding mechanism of temporin Rb with human serum albumin, holo transferrin, and hemoglobin using

spectroscopic and molecular dynamics techniques. Journal of Molecular Liquids, 389, 122833. https://doi.org/10.1016/j.molliq.2023.122833

34. Sookai, S., Bracken, M. L., & Nowakowska, M. (2023). Spectroscopic and Computational pH Study of NiII and PdII Pyrrole-Imine Chelates with Human Serum Albumin. Molecules, 28(22), 7466. https://doi.org/10.3390/molecules28227466
35. Barseem, A., Belal, F., Mabrouk, M., Hammad, S., & Ahmed, H. (2023). Probing the potential toxicity of trimetazidine by characterizing its interaction with human serum albumin. Methods and Applications in Fluorescence, 11(4), 045003. https://doi.org/10.1088/2050-6120/ace513
36. Lv, X., Li, W., Zhang, M., Wang, R., & Chang, J. (2024). Investigation of steric hindrance effect on the interactions between four alkaloids and <scp>HSA</scp> by isothermal titration calorimetry and molecular docking. Journal of Molecular Recognition. https://doi.org/10.1002/jmr.3075
37. Akbari, V., & Ghobadi, S. (2024). Evaluation of the effect of phenylpropanoids on the binding of heparin to human serum albumin and glycosylated human serum albumin concerning anticoagulant activity: A comparison study. International Journal of Biological Macromolecules, 257, 128732. https://doi.org/10.1016/j.ijbiomac.2023.128732
38. Lavanya, K., Babu, P. V., Bodapati, A. T. S., Reddy, R. S., Madku, S. R., & Sahoo, B. K. (2024). Steady-state and 3D fluorescence study reveals the binding of a dicoumarol analogue in subdomain IIA of human serum albumin with structural variation. Journal of Molecular Structure, 1298, 137032. https://doi.org/10.1016/j.molstruc.2023.137032

A1653:

1. Aseman, M. D., Aryamanesh, S., Shojaeifard, Z., Hemmateenejad, B., & Nabavizadeh, S. M. (2019). Cycloplatinated(II) Derivatives of Mercaptopurine Capable of Binding Interactions with HSA/DNA. Inorganic Chemistry, 58(23), 16154–16170. https://doi.org/10.1021/acs.inorgchem.9b02696
2. Baruah, K., Singh, A. K., Kumari, K., Nongbri, D. L., Jha, A. N., & Singha Roy, A. (2024). Interactions of Turmeric- and Curcumin-Functionalized Gold Nanoparticles with Human Serum Albumin: Exploration of Protein Corona Formation, Binding, Thermodynamics, and Antifibrillation Studies. Langmuir, 40(2), 1381–1398. https://doi.org/10.1021/acs.langmuir.3c03032
3. Tyukodi, L., Zsidó, B. Z., Hetényi, C., Kőszegi, T., Huber, I., & Rozmer, Z. (2023). Serum albumin binding studies on antiproliferative cyclic C5-curcuminoid derivatives using spectroscopic methods and molecular modelling. Journal of Molecular Structure, 1287, 135761. https://doi.org/10.1016/j.molstruc.2023.135761
4. Mohammadi, M. A., Shareghi, B., Farhadian, S., & Uversky, V. N. (2024). Investigating the effect of pH on the interaction of cypermethrin with human serum albumin: Insights from spectroscopic and molecular dynamics simulation studies. International Journal of Biological Macromolecules, 257, 128459. https://doi.org/10.1016/j.ijbiomac.2023.128459

A1887:

1. Gan, N., Sun, Q., Tang, P., Wu, D., Xie, T., Zhang, Y., & Li, H. (2019). Determination of interactions between human serum albumin and niraparib through multi-spectroscopic and computational methods. Spectrochimica Acta Part A: Molecular and Biomolecular Spectroscopy, 206, 126–134. https://doi.org/10.1016/j.saa.2018.07.100
2. Baig, M., Rahman, S., Rabbani, G., Imran, M., Ahmad, K., & Choi, I. (2019). Multi-Spectroscopic Characterization of Human Serum Albumin Binding with Cyclobenzaprine Hydrochloride: Insights from Biophysical and In Silico Approaches. International Journal of Molecular Sciences, 20(3), 662. https://doi.org/10.3390/ijms20030662
3. Rahman, S., Rehman, M. T., Rabbani, G., Khan, P., AlAjmi, M. F., Hassan, Md. I., Muteeb, G., & Kim, J. (2019). Insight of the Interaction between 2,4-thiazolidinedione and Human Serum Albumin: A Spectroscopic, Thermodynamic and Molecular Docking Study. International Journal of Molecular Sciences, 20(11), 2727. https://doi.org/10.3390/ijms20112727

4. Khatun, S., Riyazuddeen, Kumar, A., & Subbarao, N. (2020). Thermodynamics, molecular modelling and denaturation studies on exploring the binding mechanism of tetramethylpyrazine with human serum albumin. The Journal of Chemical Thermodynamics, 140, 105915. https://doi.org/10.1016/j.jct.2019.105915
5. Almutairi, F. M., Ajmal, M. R., Siddiqi, M. K., Amir, M., & Khan, R. H. (2020). Multi-spectroscopic and molecular docking technique study of the azelastine interaction with human serum albumin. Journal of Molecular Structure, 1201, 127147. https://doi.org/10.1016/j.molstruc.2019.127147
6. Mrkalić, E., Jelić, R., Stojanović, S., & Sovrlić, M. (2021). Interaction between olanzapine and human serum albumin and effect of metal ions, caffeine and flavonoids on the binding: A spectroscopic study. Spectrochimica Acta Part A: Molecular and Biomolecular Spectroscopy, 249, 119295. https://doi.org/10.1016/j.saa.2020.119295
7. Gökoğlu, E., Kıpçak, F., Taskin-Tok, T., Duyar, H., & Seferoğlu, Z. (2022). Structural analysis and calf thymus DNA/HSA binding properties of new carbazole derivative containing piperazine. Journal of Photochemistry and Photobiology A: Chemistry, 426, 113720. https://doi.org/10.1016/j.jphotochem.2021.113720
8. Zargar, S., Wani, T., Alsaif, N., & Khayyat, A. (2022). A Comprehensive Investigation of Interactions between Antipsychotic Drug Quetiapine and Human Serum Albumin Using Multi-Spectroscopic, Biochemical, and Molecular Modeling Approaches. Molecules, 27(8), 2589. https://doi.org/10.3390/molecules27082589
9. Avdović, E. H., Milanović, Ž. B., Molčanov, K., Roca, S., Vikić-Topić, D., Mrkalić, E. M., Jelić, R. M., & Marković, Z. S. (2022). Synthesis, characterization and investigating the binding mechanism of novel coumarin derivatives with human serum albumin: Spectroscopic and computational approach. Journal of Molecular Structure, 1254, 132366. https://doi.org/10.1016/j.molstruc.2022.132366
10. Fischer, F. C., Ludtke, S., Thackray, C., Pickard, H. M., Haque, F., Dassuncao, C., Endo, S., Schaider, L., & Sunderland, E. M. (2024). Binding of Per- and Polyfluoroalkyl Substances (PFAS) to Serum Proteins: Implications for Toxicokinetics in Humans. Environmental Science & Technology, 58(2), 1055–1063. https://doi.org/10.1021/acs.est.3c07415
11. Poddar, S., Woolfork, A. G., Iftekhar, S., Ovbude, S. T., & Hage, D. S. (2023). Characterization of binding by sulfonylureas with normal or modified human serum albumin using affinity microcolumns prepared by entrapment. Journal of Chromatography B, 1226, 123798. https://doi.org/10.1016/j.jchromb.2023.123798
12. Rahman, S., Iram, S., Rehman, M. T., Hussain, A., Jan, A. T., & Kim, J. (2023). Study of Amiloride Binding to Human Serum Albumin: Insights from Thermodynamic, Spectroscopic, and Molecular Docking Investigations. Molecules, 28(23), 7688. https://doi.org/10.3390/molecules28237688
13. Asngari, N. J. M., Bakar, K. A., Feroz, S. R., Razak, F. A., & Halim, A. A. A. (2024). Interaction mechanism of a cysteine protease inhibitor, odanacatib, with human serum albumin: In vitro and bioinformatics studies. Biophysical Chemistry, 305, 107140. https://doi.org/10.1016/j.bpc.2023.107140

A3782:

1. Tayyab, S., Francis, J. A., Kabir, Md. Z., Ghani, H., & Mohamad, S. B. (2019). Probing the interaction of 2,4-dichlorophenoxyacetic acid with human serum albumin as studied by experimental and computational approaches. Spectrochimica Acta Part A: Molecular and Biomolecular Spectroscopy, 207, 284–293. https://doi.org/10.1016/j.saa.2018.09.033
2. Chaves, O. A., Sasidharan, R., dos Santos de Oliveira, C. H. C., Manju, S. L., Joy, M., Mathew, B., & Netto-Ferreira, J. C. (2019). In Vitro Study of the Interaction Between HSA and 4-Bromoindolylchalcone, a Potent Human MAO-B Inhibitor: Spectroscopic and Molecular Modeling Studies. ChemistrySelect, 4(3), 1007–1014. https://doi.org/10.1002/slct.201802665
3. Tayyab, S., Sam, S. E., Kabir, Md. Z., Ridzwan, N. F. W., & Mohamad, S. B. (2019). Molecular interaction study of an anticancer drug, ponatinib with human serum albumin using

spectroscopic and molecular docking methods. Spectrochimica Acta Part A: Molecular and Biomolecular Spectroscopy, 214, 199–206. https://doi.org/10.1016/j.saa.2019.02.028

4. Yasrebi, S. A., Takjoo, R., & Riazi, G. H. (2019). HSA-interaction studies of uranyl complexes of alkyl substituted isothiosemicarbazone. Journal of Molecular Structure, 1193, 53–61. https://doi.org/10.1016/j.molstruc.2019.04.126
5. Moriyama, Y., & Takeda, K. (2020). Removal of Dodecyl Sulfate Ions Bound to Human and Bovine Serum Albumins Using Sodium Cholate. Journal of Oleo Science, 69(1), 65–72. https://doi.org/10.5650/jos.ess19224
6. Kabeer, H., Hanif, S., Arsalan, A., Asmat, S., Younus, H., & Shakir, M. (2020). Structural-Dependent N,O-Donor Imine-Appended Cu(II)/Zn(II) Complexes: Synthesis, Spectral, and in Vitro Pharmacological Assessment. ACS Omega, 5(2), 1229–1245. https://doi.org/10.1021/acsomega.9b03762
7. Yang, H., Zeng, Q., He, Z., Wu, D., & Li, H. (2020). Interaction of novel Aurora kinase inhibitor MK-0457 with human serum albumin: Insights into the dynamic behavior, binding mechanism, conformation and esterase activity of human serum albumin. Journal of Pharmaceutical and Biomedical Analysis, 178, 112962. https://doi.org/10.1016/j.jpba.2019.112962
8. Yekke-ghasemi, Z., Ramezani, M., Mague, J. T., & Takjoo, R. (2020). Synthesis, characterization and bioactivity studies of new dithiocarbazate complexes. New Journal of Chemistry, 44(21), 8878–8889. https://doi.org/10.1039/D0NJ01187H
9. Musa, K. A., Ning, T., Mohamad, S. B., & Tayyab, S. (2020). Intermolecular recognition between pyrimethamine, an antimalarial drug and human serum albumin: Spectroscopic and docking study. Journal of Molecular Liquids, 311, 113270. https://doi.org/10.1016/j.molliq.2020.113270
10. Ali, M. S., Rehman, M. T., Al-Lohedan, H., & AlAjmi, M. F. (2022). Spectroscopic and Molecular Docking Investigation on the Interaction of Cumin Components with Plasma Protein: Assessment of the Comparative Interactions of Aldehyde and Alcohol with Human Serum Albumin. International Journal of Molecular Sciences, 23(8), 4078. https://doi.org/10.3390/ijms23084078
11. Alizadeh, S., Mague, J. T., & Takjoo, R. (2022). Structural, theoretical investigations and HSA-interaction studies of three new copper(II) isothiosemicarbazone complexes. Polyhedron, 224, 115986. https://doi.org/10.1016/j.poly.2022.115986
12. Kubczak, M., Grodzicka, M., Michlewska, S., Karimov, M., Ewe, A., Aigner, A., Bryszewska, M., & Ionov, M. (2023). The effect of novel tyrosine-modified polyethyleneimines on human albumin structure – Thermodynamic and spectroscopic study. Colloids and Surfaces B: Biointerfaces, 227, 113359. https://doi.org/10.1016/j.colsurfb.2023.113359
13. Sajid Ali, M., Singh, E., Muthukumaran, J., & Al-Lohedan, H. A. (2023). Non-Steroidal Anti-Inflammatory Drug Effect on the Binding of Plasma Protein with Antibiotic Drug Ceftazidime: Spectroscopic and In Silico Investigation. International Journal of Molecular Sciences, 24(19), 14811. https://doi.org/10.3390/ijms241914811
14. Abolhassan, M. R., Divsalar, A., Badalkhani-khamseh, F., Kheiripour, N., Eslami-Moghadam, M., & Mirzaei, H. (2023). Protein binding and anticancer activity of two newly synthesized Schiff base platinum (II) complexes: A theoretical and experimental study. Journal of Molecular Structure, 1289, 135917. https://doi.org/10.1016/j.molstruc.2023.135917
15. Chaves, O. A., dos Santos Oliveira, C. H. C., Ferreira, R. C., Cesarin-Sobrinho, D., Machado, A. E. da H., & Netto-Ferreira, J. C. (2024). Synthetic dimethoxyxanthones bind similarly to human serum albumin compared with highly oxygenated xanthones. Chemical Physics Impact, 8, 100411. https://doi.org/10.1016/j.chphi.2023.100411

A8763:

1. Dömötör, O., & Enyedy, É. A. (2019). Binding mechanisms of half-sandwich Rh(III) and Ru(II) arene complexes on human serum albumin: a comparative study. JBIC Journal of Biological Inorganic Chemistry, 24(5), 703–719. https://doi.org/10.1007/s00775-019-01683-0

A9511:

1. M., M., & H.D., R. (2019). Interpretation of the binding interaction between bupropion hydrochloride with human serum albumin: A collective spectroscopic and computational approach. Spectrochimica Acta Part A: Molecular and Biomolecular Spectroscopy, 209, 264–273. https://doi.org/10.1016/j.saa.2018.10.047
2. Yang, Y.-D., Lu, N., & Tian, R. (2023). Serum albumin acted as an effective carrier to improve the stability of bioactive flavonoid. Amino Acids, 55(12), 1879–1890. https://doi.org/10.1007/s00726-023-03347-5
3. Senft, M. D., Maier, R., Hiremath, A., Zhang, F., & Schreiber, F. (2023). Effective interactions and phase behavior of protein solutions in the presence of hexamine cobalt(III) chloride. The European Physical Journal E, 46(12), 119. https://doi.org/10.1140/epje/s10189-023-00376-6

A9731:

1. Vaneková, Z., Hubčík, L., Toca-Herrera, J., Furtműller, P., Valentová, J., Mučaji, P., & Nagy, M. (2019). Study of Interactions between Amlodipine and Quercetin on Human Serum Albumin: Spectroscopic and Modeling Approaches. Molecules, 24(3), 487. https://doi.org/10.3390/molecules24030487
2. Zhang, H., Zhang, T., & Wang, Y. (2019). Mechanistic understanding and binding analysis of two-dimensional MoS2 nanosheets with human serum albumin by the biochemical and biophysical approach. Spectrochimica Acta Part A: Molecular and Biomolecular Spectroscopy, 211, 18–25. https://doi.org/10.1016/j.saa.2018.11.055
3. Vaneková, Z., Hubčík, L., Toca-Herrera, J. L., Furtműller, P. G., Mučaji, P., & Nagy, M. (2020). Analysis of Binding Interactions of Ramipril and Quercetin on Human Serum Albumin: A Novel Method in Affinity Evaluation. Molecules, 25(3), 547. https://doi.org/10.3390/molecules25030547

SRP6182:

1. Zhang, S., Gan, R., Zhao, L., Sun, Q., Xiang, H., Xiang, X., Zhao, G., & Li, H. (2021). Unveiling the interaction mechanism of alogliptin benzoate with human serum albumin: Insights from spectroscopy, microcalorimetry, and molecular docking and molecular dynamics analyses. Spectrochimica Acta Part A: Molecular and Biomolecular Spectroscopy, 246, 119040. https://doi.org/10.1016/j.saa.2020.119040
2. Singh, S., Gopi, P., & Pandya, P. (2022). Structural aspects of formetanate hydrochloride binding with human serum albumin using spectroscopic and molecular modeling techniques. Spectrochimica Acta. Part A, Molecular and Biomolecular Spectroscopy, 281, 121618. https://doi.org/10.1016/j.saa.2022.121618
3. Azeem, K., Ahmed, M., Uddin, A., Singh, S., Patel, R., & Abid, M. (2023). Comparative investigation on interaction between potent antimalarials and human serum albumin using multispectroscopic and computational approaches. Luminescence, 38(12), 2018–2033. https://doi.org/10.1002/bio.4590
4. Chamlagai, D., Bora, P., Bhatta, A., Upadhyaya, J., Phanrang, P. T., Bora, U., & Mitra, S. (2024). Donor-acceptor functionalized coumarin derivatives: Synthesis, fluorescence modulation, interaction with human serum albumin and acetylcholinesterase inhibition activity. Journal of Photochemistry and Photobiology A: Chemistry, 447, 115273. https://doi.org/10.1016/j.jphotochem.2023.115273
